# Albumin Nanocages with Methotrexate and Chondroitin Sulfate as a Dual pH/GSH-Responsive Tumor Targeting Nanomedicine for Synergistic Cancer Therapy

**DOI:** 10.34133/bmr.0245

**Published:** 2025-09-03

**Authors:** Haroon Iqbal, Anam Razzaq, Ziyin Yuan, Lina Zhai, Yue Wang, Uzair Ur-Rehman, Lv Man, Jun Xin, Xin Ning, Yuanbo Liang, Run Xiao

**Affiliations:** ^1^ Zhejiang Cancer Hospital, Hangzhou Institute of Medicine, Chinese Academy of Sciences, Hangzhou, Zhejiang 310022, China.; ^2^ Eye Research Center, Hangzhou Institute of Medicine, Chinese Academy of Sciences, Eye Hospital, Wenzhou Medical University, Hangzhou, 310018, China.; ^3^ Jiangsu Key Laboratory of Neuropsychiatric Diseases, College of Pharmaceutical Science, Soochow University, Suzhou, 215123, China

## Abstract

Cancer is a devastating disease, and its pathogenesis is highly associated with malnutrition and poor lifestyle. Chemotherapy continuously causes inadequate therapeutic efficacy and induces off-target toxicities. Hence, targeted co-administration of chemotherapy and dietary supplement producing anticancer effect at low doses with minimized toxicities would be a promising strategy for cancer treatment. In this study, we constructed chondroitin sulfate (CS) and methotrexate (MTX) carried serum albumin nanocages (C/M@Alb NCs) by albumin nanoreactor strategy. During fabrication, we achieved the precipitation of MTX and CS inside the albumin nanocore under mild reaction condition to prepare C/M@Alb NCs. The enhanced anticancer efficacy of C/M@Alb NCs was comprehensively assessed by in vitro and in vivo experiments. Biodistribution, pharmacokinetic profile, and in vivo therapeutic efficacy of C/M@Alb NCs were investigated in human colorectal adenocarcinoma (HT-29), murine breast cancer (E0071), and patient-derived (PDX) lung cancer models. The as-prepared C/M@Alb NCs facilitated higher MTX and CS encapsulation, exhibiting small particle size, improved colloidal stability, dual stimuli (pH/GSH)-responsive drug release profile, an enhanced cellular uptake, cooperative synergistic cytotoxicity, extended blood residence time, improved lymph node and tumor targeting, and in vivo therapeutic efficacy against various cancers such as human colorectal adenocarcinoma, murine breast cancer, and patient-derived (PDX) lung cancer. Altogether, C/M@Alb NCs exhibited enhanced cellular uptake, extended blood residence time, and favorable tumor accumulation and lymph node extravasation, finally leading to the potent antitumor efficacy against various cancers. This nanoplatform offers a new strategy for designing lymph node- and cancer-targeted albumin-based nanomedicine for clinical applications.

## Introduction

Cancer still threatens the human life with severe mortality rate among those under the age of 85 years [1]. Currently, anticancer agents such as doxorubicin, irinotecan, cisplatin, 5-fluorouracil, and temozolomide are used as a first-line cancer treatment to induce cell apoptosis and inhibit the tumor cell growth; however, the therapeutic potential of these anticancer agents is inadequate due to severe off-target side effects, extensive distribution to the healthy tissues, and development of chemoresistance [2–4]. Hence, the development of a new therapeutic strategy to effectively inhibit the growth of cancer cells with minimal doses is highly demanding.

Malnutrition or unhealthy diet is presumed to facilitate the cancer cell growth, so the application of dietary supplement with an anticancer activity might be an encouraging therapeutic approach for cancer treatment [2]. Chondroitin sulfate (CS) has been exploited as a multipurpose food supplement and nutraceutical with proficient activities, such as regulation of cell growth, integrity of tissue structure, anti-arthritic, anti-inflammatory, and anticancer activity, which might be reflected as a promising therapeutic agent for cancer management [2,5–7]. CS as an anticancer agent has attracted a substantial attention of the researchers in recent years. For example, sturgeon CS repressed the growth of colon cancer cells by triggering the mitochondrial apoptotic pathway [5]. In another study, Wu and Shang [8] described the enhanced in vitro and in vivo anticancer activity of CS against colorectal cancer. Thus, we theorized that the anticancer efficacy of CS might be improved by employing as combination therapy.

Methotrexate (MTX) is a selective folate antagonist, blocks the enzymatic activity of dihydrofolate reductase, and thus inhibits the translation of tetrahydrofolate from dihydrofolate, which in turn stops the synthesis of thymidylates and nucleic acids (RNA and DNA) and causes cell death [9–12]. However, MTX’s poor water solubility, short systemic circulation time, low cellular uptake, imprecise tissue distribution, and severe off-target side effects restrict its chemotherapeutic efficacy [13–15]. Henceforward, these limitations have endorsed the researchers to develop a targeted therapeutic nanoplatform with negligible toxicities to healthy cells/tissues.

Albumin has been extensively explored as nanocarriers for precise tumor targeting due to their extended blood circulation (~19 d) and heightened tumor accumulation ability [16–21]. Furthermore, albumin is recruited by the tumor cells via albumin receptors such as SPARC (osteonectin) and gp60 (albondin) highly expressed on tumor cells; additionally, the aggressive tumor cells exploited albumin protein as a food and nutrients source to satisfy their needs for growth [16,22,23]. Moreover, albumin-based nanoparticles (NPs) with small particle size have high extravasation tendency to lymphatic system, especially appropriate for lymph node (LN) targeting drug nanocarrier [24]. Up to now, albumin-based nanomedicine with paclitaxel (Abraxane) has been successfully approved for clinical use, which markedly improved the therapeutic efficiency of paclitaxel with negligible off-target side effects [25,26].

Albumin nanoreactor strategy has been extensively investigated for the synthesis of organic drug-loaded albumin NPs [27,28]. Still, it is challenging to employ this strategy for organic small drug molecules such as MTX to be precipitated within albumin nanocavity and further developed drug-loaded albumin NPs with active tumor targeting capability. In the present study, we construct albumin nanocages via albumin nanoreactor method by reacting preactivated CS with MTX inside the hallow albumin nanocavity. In this process, preactivated CS reacted with MTX via precipitation reaction within the albumin nanocage and further grew to CS-MTX albumin nanocages (C/M@Alb NCs) under optimized conditions. The prepared C/M@Alb NCs exhibited various advantages such as small PS, apt drug loading capacity, greater cellular uptake, dual pH/GSH-responsive drug release, higher intracellular reactive oxygen species (ROS) scavenging ability, extended systemic circulation time, precise tumor targeting, LN accumulation, and a first-rate biosafety. C/M@Alb NCs unveiled synergistic in vivo therapeutic efficacy against various tumors such as HT-29 subcutaneous colorectal tumors, E0771 orthotopic murine breast tumors, and patient-derived lung tumors, indicating a potential nanotherapeutic agent for the treatment of several types of cancers in the clinic.

## Materials and Methods

### Materials

CS [(CH_14_H_21_NO_14_S)*_n_*, chondroitin polysulfate] and methotrexate hydrate were obtained from MedChemExpress (Shanghai, China). Human serum albumin (HSA) was supplied by Sino Biological (Bejing, China). 4′,6-Diamidino-2-phenylindole (DAPI) and LysoTracker Red were acquired from KeyGen Biotech (Nanjing, China). All other chemicals reagents with analytical grade were used during the project.

HT-29 colon cancer line was provided by Meissen Cell Technology (Hangzhou, China). The cell line was grown in Ham nutrient (F-12) mixed Dulbecco’s modified Eagle’s medium (DMEM) (DMEM/F-12) supplied with fetal bovine serum (FBS) (10%) and penicillin–streptomycin solution (1%, v/v) in 5% CO_2_ incubator. E0071 breast cancer line was provided by Nanjing University Cell Bank (Nanjing, China), and further, the cell line was grown in RPMI 1640 supplemented with FBS (10%) and penicillin–streptomycin solution (1%, v/v) in 5% CO_2_ incubator.

Nude BALB/c (male and female) and C57BL/6 (female) mice were obtained from Zhejiang Experimental Animal Center (Zhejiang, China); they were treated according to the guidelines provided by Hangzhou Institute of Medicine, Chinese Academy of Science Animal Center (approval no.: 2023R0027).

### Preparation of C/M@Alb NCs

C/M@Alb NCs were prepared by precipitation reaction between preactivated CS and MTX within albumin core via albumin nanoreactor strategy [27]. Briefly, HSA solution (10 mg ml^−1^) was prepared in distilled water (dH_2_O) followed by the addition of 1.0 ml preactivated CS solution (8.8 mM) under continuous mixing. After continuous stirring for 10 min, 1.0 ml of MTX solution (2.2 mM) was gently supplied and the pH of solution was adjusted to 7.00 by NaOH solution (0.5%, v/v). Afterward, the reaction mixture was grown at 37 ± 2 °C for 4 h under continuous stirring (500 rpm). Finally, the prepared suspension was centrifuged (7,000 rpm, 10 min) to remove the free reagents and the supernatant was further purified using centrifugal ultrafiltration units (Millipore, molecular weight cutoff = 100 kDa) at 2,000 rpm for 10 min (5 cycles) and the obtained suspension of C/M@Alb NCs was placed at 4 °C for subsequent use(s).

### Physicochemical characterizations of C/M@Alb NCs

The hydrodynamic diameter of PS, the particle size distribution (PSD), and the zeta potential (ZP) of C/M@Alb NCs were assessed by Zetasizer (Malvern, Zetasizer Nano ZS90, UK). Further, the core diameter and morphology of C/M@Alb NCs were analyzed by transmission electron microscopy (TEM; JEM-2100plus, Japan). Fourier transform infrared (FTIR) spectrum of C/M@Alb NCs was recorded and compared with free CS and MTX and has spectra to examine eventual drug–protein interactions and unravel functional groups. The percent drug loading capacity (LC %) and encapsulation efficiency (EE %) of C/M@Alb NCs were measured in 1× phosphate-buffered saline (PBS) (pH 7.4) using a multimode microplate reader (Spark, Tecan AG, Switzerland) at 304 nm for MTX by calibration curve and CS enzyme-linked immunosorbent assay (ELISA) kit for CS quantification. The % LC and EE were calculated as follows:

$$\mathrm{LC}\;\left(\%\right)=\frac{\mathrm{CS}/\mathrm{MTX}\;\text{in}\;\mathrm{NPs}}{\text{Weight of dried}\;\mathrm{NPs}}\times 100$$ (1)

$$\mathrm{EE}\;\left(\%\right)=\frac{\mathrm{CS}/\mathrm{MTX}\;\text{in}\;\mathrm{NPs}}{\mathrm{CS}/\mathrm{MTX}\;\text{initially added}}\times 100$$ (2)

Next, we analyzed the nature of drugs after encapsulating into C/M@Alb NCs by x-ray diffractometer (XRD; Bruker D8 Advance Diffractometer, Germany). Moreover, the albumin secondary structure in C/M@Alb NCs was analyzed by circular dichroism spectrometer (Lakewood, NJ, USA). The dispersion stability was evaluated by incubating C/M@Alb NCs in different dissolution medium [DMEM/F12 with 10% FBS, 0.9% NaCl, dH_2_O, 8.0% glucose, and 1× PBS (pH 7.4)] at 4 °C. Afterward, at specified time periods (0, 6, 12, 24, 48, 96, and 120 h), the ZP and hydrodynamic PS were measured by Zetasizer. Finally, we evaluated the accelerated temperature stability of C/M@Alb NCs. For this, freshly prepared lyophilized C/M@Alb NCs were stored for 21 d at temperatures of −20, 4, 25, and 37 °C. After storing, C/M@Alb NCs were analyzed for visual changes in color, morphology, PS, and drug content.

### In vitro drug release studies

To study the release behavior of MTX from C/M@Alb NCs, in vitro drug release study was performed at pH 7.4 (normal physiological) and pH 5.0 (tumor microenvironment) and with low (2.0 μM, extracellular matrix) and high (10.0 mM, tumor cell cytosol environment) concentration of GSH. Briefly, 1 ml of C/M@Alb NC suspension (0.5 mg ml^−1^ MTX, 0.7 mg ml^−1^ CS) was placed in a tightly sealed dialysis bag (3.5 kDa) and then immersed into 15-ml tube supplied with acetate buffer (pH 5.0) and 1× PBS (pH 7.4) solution with or without GSH and stirred (120 rpm) at 37 ± 2 °C. At predetermined time intervals (0, 0.5, 1.0, 2.0, 4.0, 8.0, 12.0, and 24.0 h), 2.0 ml of aliquot was withdrawn from release medium and equal volume of fresh release medium was quickly supplied to each tube. The respective concentration of MTX and CS released from C/M@Alb NCs was determined by microplate reader (Switzerland, Spark, Tecan AG) at 304 nm for MTX using calibration curve and ELISA kit for CS; the percent cumulative release of MTX and CS from C/M@Alb NCs was calculated. The drug release experiment was carried out in triplicate independently, and the results were reported as mean value ± standard deviation (SD).

The obtained drug release data were fitted to the Peppas–Sahlin model to understand the mechanism underlying the release of MTX from C/M@Alb NCs using Eq. 3. The Peppas–Sahlin model describes the release from a drug delivery system, where 2 simultaneous release phenomena, such as diffusion and relaxation of albumin chains, occur.

$$\frac{\mathrm{Mt}}{\;M\infty }=\mathrm{Mt}\;k1.t^{n}+k2.t^{2n}$$ (3)

where *Mt*/*M*∞ ration denotes the cumulative release of MTX at a certain time *t*, *k*1 first kinetic constant signifies the Fickian diffusion contribution to the release mechanism, *k*2 second kinetic constant suggests the involvement of the albumin chain relaxation in drug release, and *n* reveals the release mechanism of drug delivery system.

### Cellular uptake studies

To examine cellular uptake, HT-29 cells were cultured in 6-well plates (1.0 × 10^5^ cells per well) and grew overnight in 5% CO_2_ at 37 °C. Next, the cells were incubated with C/M@Alb NCs or MTX (control) for a specific time (2, 6, 12, or 24 h) at the fixed concentration of 2.0 μg ml^−1^ MTX or for a fixed time point (24 h) at different concentrations of MTX (0.5 to 2.0 μg ml^−1^) to determine the time- and concentration-dependent cellular uptake. Subsequently, the cells were carefully washed and collected after detaching with trypsin (0.25%); the cells were resuspended in 1× PBS (pH 7.4) and then accurately counted using an automatic cytometer (Invitrogen, China). Next, the cells were crushed by an ultra-probe sonication method. The cell suspensions were centrifuged, and the supernatants were collected. Eventually, the amount of MTX in the supernatant was measured by multimode microplate reader (Switzerland, Spark, Tecan AG) at 304 nm for MTX using calibration curve.

### Mechanism of cellular uptake

To examine the cell internalization mechanism or pathway, HT-29 cells (1 × 10^5^ cells per well) were seeded in 6-well plates for overnight growth. Subsequently, they were incubated for 1 h with various inhibitors such as chlorpromazine (10.0 μg ml^−1^, inhibitor of clathrin-dependent endocytosis), amiloride (100.0 μg ml^−1^, inhibitor of macropinocytosis), and nystatin (5.0 μg ml^−1^, inhibitor of caveolin-dependent endocytosis). Then, C/M@Alb NCs (5.0 μg ml^−1^ CS, 2.0 μg ml^−1^ MTX) were added to the medium for 6-h incubation; to distinguish active and passive transport, the incubation occurred at 4 and 37 °C, respectively. To determine the albumin receptor-induced endocytosis, HT-29 cells were pretreated with albumin before exposure to C/M@Alb NC treatment. Afterward, cells were washed, trypsinized, collected, counted, and ruptured under ultra-probe sonication. Finally, the concentration of MTX and CS in the supernatant was measured by multimode microplate reader (Switzerland, Spark, Tecan AG) at 304 nm for MTX using calibration curve.

### Intracellular distribution

To evaluate the intracellular distribution of C/M@Alb NCs, HT-29 cells were cultivated in glass-bottom confocal plates at a cell density of 1 × 10^5^ cells per plate for overnight growth. They were further incubated with fluorescein isothiocyanate (FITC)-tagged C/M@Alb NCs for 12 h. Subsequently, the cells were aspirated, washed thrice with 1× PBS (pH 7.4), and stained with LysoTracker Red DND-99 (100 nM) for 10 min, followed by DAPI (5.0 μg ml^−1^) staining for 5 min in the dark. Finally, the florescence photographs were obtained by confocal laser scanning microscopy (CLSM) (Zeiss LSM710).

### In vitro antitumor efficacy and synergistic effect evaluation

The in vitro antitumor efficacy of C/M@Alb NCs was assessed by cell counting kit-8 (CCK-8) assay. Briefly, HT-29 cells (1 × 10^4^ cells) were seeded in each well of 96-well plate supplied with specific growth medium (DMEM-F12) and were incubated overnight. Subsequently, the cells were cautiously aspirated and carefully washed thrice with 1× PBS (pH 7.4) before fresh medium containing different concentrations of CS, MTX, CS/MTX, and C/M@Alb NCs was supplied to the cells that were further incubated for 24 and 48 h (*n* = 3). Subsequently, the percent (%) growth inhibition induced by the treatment regimen was measured by CCK-8 cell counting kit following the manufacturer’s protocol.

The synergism between MTX and CS loaded into C/M@Alb NCs was evaluated in HT-29 cells using isobologram analysis. Briefly, the dose–response curves of CS and MTX in HT-29 cells were first generated independently. The synergism between CS and MTX in C/M@Alb NCs was then determined by combination index (CI) by following the below isobologram equation:

$$\mathrm{CI}=\frac{D1}{d1}+\frac{D2}{d2}$$ (4)

where *D*1 and *D*2 are the corresponding concentrations of CS and MTX in C/M@Alb NCs, respectively, required to induce a specified level of growth inhibition; *d*1 and *d*2 are the concentrations of free CS and free MTX able to induce the same inhibitory effect alone. CI value <1 signifies that there is a synergistic effect between the 2 drugs, whereas CI value greater or equal to 1 indicates that the interaction is antagonistic or additive, respectively.

### Apoptosis assay

Apoptosis induced by C/M@Alb NCs in HT-29 cells was evaluated by using annexin V–FITC/DAPI staining assay. HT-29 cells (1 × 10^5^ cells/well) were seeded in 6-well plates and incubated overnight. Subsequently, the cells were washed with 1× PBS (pH 7.4) and treated with CS, MTX, CS/MTX, and C/M@Alb NCs for 24 h (*n* = 3). After 24-h incubation, the cells were washed, trypsinized (0.25% trypsin without EDTA), and centrifuged at 3,000 rpm and 4 °C for 5 min; the pellet (after discarding the supernatant) was resuspended in 200 μl of binding buffer, followed by staining with DAPI and annexin V–FITC. The mixture was further incubated for 0.5 h at 37 °C in a light-protected incubator. Finally, the stained cells were analyzed by FACScan flow cytometry system (BD LSR Fortessa, NJ, USA) and processed by FlowJo v 0.7 software (BD Biosciences, Ashland, NJ, USA).

### Cell cycle analysis

For cell cycle study, HT-29 cells (1 × 10^5^ cells/well) were cultured in 6-well plates and grown for 12 h. Afterward, the cells were aspirated, washed with 1× PBS (pH 7.4), and incubated with CS, MTX, CS/MTX, and C/M@Alb NCs for 24 h (*n* = 3). After treatment, cells were detached with 0.25% trypsin without EDTA and collected by centrifugation at 1,000 rpm and 4 °C for 5 min. Next, the cell pellets were resuspended in prechilled 1× PBS (pH 7.4) for washing followed by centrifugation at 1,000 rpm and 4 °C for 5 min. After centrifugation, the harvested cell pellets were fixed with prechilled ethanol solution (70%) at 4 °C for 24 h and carefully washed twice with prechilled 1× PBS (pH 7.4) before they were resuspended in 535 μl of staining solution (comprising 500 μl of staining buffer, 10 μl of ribonuclease A, and 25 μl of propidium iodide) and incubated at 37 °C for 30 min in the dark. Ultimately, the cells were processed by the flow cytometry system (BD LSR Fortessa, NJ, USA) and processed by FlowJo v 0.7 software (BD Biosciences, Ashland, NJ, USA).

### Pharmacokinetic studies

To analyze the pharmacokinetic profile, healthy male ICR (with background of BALB/c) aged 6 to 7 weeks with an average body weight of 16 ± 2 g (*N* = 9) were separated into 2 groups (*n* = 3 per group), that is, free MTX group (10.0 mg kg^−1^ body weight) and C/M@Alb NC group (10.0 mg kg^−1^ MTX body weight), and fed with balanced food. At predetermined time points (0.1, 1.0, 3.0, 6.0, 12.0, 24.0, 36.0, and 48.0 h), the blood samples (150 ± 50 μl) were carefully collected by retroorbital bleeding into heparin-coated epi-tubes, followed by centrifugation at 13,000 rpm and 4 °C for 20 min. Subsequently, the plasma concentration of MTX was quantified in all samples by multimode microplate reader (Spark, Tecan AG, Switzerland). The time-dependent concentration of MTX in the plasma was calculated using a standard curve, generated by mixing the known amount of MTX in free plasma (attained from untreated ICR mice). Plasma from PBS-treated mice was applied as a control group. The plasmatic concentration of MTX versus time curve was produced; the subsequent area under the curve (AUC) and the biological half-life (*t*_1/2_) of MTX were calculated by Kinetica 4.4 (Waltham, MA, USA).

We also collected the blood sample (100 μl) from all mice by retroorbital bleeding injected with Ce6-conjugated C/M@Alb NCs and free Ce6 (3.5 mg kg^−1^ with respect to Ce6) at predetermined time points (0.1, 1.0, 3.0, 6.0, 12.0, 24.0, 36.0, and 48.0 h), using a sterile heparin-coated capillary tube. Afterward, the blood samples were added to the detergent solution [deionized water (DW):PBS:dimethyl sulfoxide (DMSO) at 1:4:5, Triton X-100, 1%] at a 9:1 (v/v) blood/detergent ratio, and the fluorescence signals of Ce6 were detected by IVIS Lumina III.

### Biodistribution of C/M@Alb NCs

To examine the tumor distribution of C/M@Alb NCs, Ce6-conjugated C/M@Alb NCs and free Ce6 (3.5 mg kg^−1^ with respect to Ce6) were intravenously injected to HT-29 tumor-bearing BALB/c male mice (*n* = 3). At 24 h post-injection, all mice were forfeited by cervical displacement (approved sacrificing method for small animals), and the major tissues (i.e., liver, heart, spleen, kidney, and lungs), tumors, nonsentinel lymph node (NSLN), and sentinel lymph node (SLN) around the tumors were excised and analyzed by IVIS Lumina III. Afterward, 0.2 g of each extracted tissue was precisely weighed and crushed in 500 μl of 1× PBS (pH 7.4) using a high-speed homogenizer (BAOSHISHAN FS-600N, CA, USA), supplied with 1.0 ml of detergent solution (DW:PBS:DMSO at 1:4:5, Triton X-100, 1%) to extract the MTX and CS from tissues, and centrifuged at 10,000 rpm for 10 min. The upper layer of the supernatant was carefully collected, and the MTX and CS concentrations were then quantified by multimode microplate reader (Spark, Tecan AG, Switzerland). The MTX and CS concentrations were calculated by using a calibration curve accomplished by using the known amount of MTX and CS ELISA kit.

### Maximum tolerated dose analysis

To estimate the maximum therapeutic dose of C/M@Alb NCs tolerated by the mice, healthy C57BL/6 (Black 6) mice (*N* = 30, *n* = 5 per group) were intravenously injected with free MTX and C/M@Alb NCs via vein tail injection at different doses (i.e., 10.0, 20.0, and 30.0 mg kg^−1^ MTX). The behavioral changes, survival rate, and body weight of mice were monitored during the 3 weeks post-injection.

### Subcutaneous HT-29 xenograft model

BALB/c nude mice (*N* = 25, male) aged 8 to 10 weeks with a median body weight of 22 ± 2 g were separated into 5 groups (*n* = 5 mice per cage) and housed with a 12-h dark/12-h light cycle at 37 ± 2 °C under 60 ± 5% humidity. Food and water were available ad libitum throughout this study. All the experimental procedures were checked and permitted by the Research and Animal Ethics Committee of Hangzhou Institute of Medicine, Chinese Academy of Science, Hangzhou, China.

A subcutaneous HT-29 xenograft model was stimulated in BALB/c nude mice using a reported method with some modifications [2]. Briefly, HT-29 cells (1 × 10^7^ cells) suspended in 100 μl of 1× PBS (pH 7.4) were subcutaneously injected to the right thigh of all mice and the tumor growth was observed daily. When the tumor volumes reached 50 ± 10 mm^3^ in diameter, HT-29 tumor-induced mice were randomly divided into 5 groups (*n* = 5 mice per group) with the following treatments: (a) control group: tumor-induced mice with intravenous administration of PBS (5.0 μl g^−1^ body weight/day); (b) CS group: tumor-induced mice with intravenous administration of CS (100.0 mg kg^−1^ body weight/day); (c) MTX group: tumor-induced mice with intravenous administration of MTX (10.0 mg kg^−1^ body weight/day); (d) CS-MTX group: tumor-induced mice with intravenous administration of CS/MTX (100.0 mg kg^−1^ of CS and 10.0 mg kg^−1^ body weight/day of MTX); (e) C/M@Alb NC group: tumor-induced mice with intravenous administration of C/M@Alb NCs (≈100.0 mg kg^−1^ and 10.0 mg kg^−1^ body weight/day). The tumor volume and body weight were measured every third day by vernier caliper and electrical weighing balance, respectively. Subsequently, the volume of each tumor was calculated using the following formula:

$$\text{Tumor}\;\text{volume}=\frac{\text{length}\times {\text{width}}^{2}}{2}$$ (5)

The mice in each cage were monitored daily for their survival during the experimental period. At the end of experimental period (21 d), all the mice were sacrificed [according to the replacement, reduction, and refinement (3R) rules and the cervical dislocation method approved for small animals], and the tumors were harvested, weighted, and photographed. The freshly excised tumors and all major organs were washed with ice-cold 1× PBS (pH 7.4) and then fixed with 4% paraformaldehyde, embedded, sliced, and stained with hematoxylin and eosin (H&E).

### Orthotopic E0771 breast tumor model

In a further step, we estimated the antitumor efficacy and lung metastasis inhibition of C/M@Alb NCs using the orthotopic E0771 breast tumor model. Thereby, 1 × 10^7^ E0771 tumor cells were injected into the mammary pad of female C57/BL6 mice (*N* = 25), and the tumor growth was monitored. When tumors achieved the size of about 70 ± 10 mm^3^, the mice were randomly segregated into 5 groups (*n* = 5) and injected with PBS (5.0 μl g^−1^), free MTX (10.0 mg kg^−1^), free CS (100.0 mg kg^−1^), CS-MTX (CS: ≈100.0 mg kg^−1^ and MTX: 10.0 mg kg^−1^), and C/M@Alb NCs (CS: ≈100.0 mg kg^−1^ and MTX:10.0 mg kg^−1^) at days 7, 9, and 11 post-tumor inoculation. The tumor volumes and body weight were measured every third day during the next 21 d and the tumor volume was calculated by Eq. 4 as previously mentioned. On day 21, d-luciferin aqueous solution (5.0 mg/ml) was injected in peritoneal cavity 15 min prior to the sacrificing of the mice, which were subsequently dissected to harvest the lungs for bioluminescence imaging (Caliper Life Sciences, USA).

### Patient-derived lung tumor model

Further, the antitumor efficacy of C/M@Alb NCs was valiadted in PDX lung tumor model. Briefly, a small piece (~60 to 80 mm^3^) of PDX was implanted into the right pectoral area of each female nude (immunodeficient) BALB/c mice aged 4 to 5 weeks using a biopsy trocar needle. Tumor growth was visualized daily until the palpable stage and their sizes were then measured by vernier caliper. When the tumors grow to 70 ± 10 mm^3^ sizes, mice were separated into 5 groups (*n* = 5) injected with PBS (150 μl), free MTX (10.0 mg kg^−1^), free CS (100.0 mg kg^−1^), CS-MTX (CS: ≈100.0 mg kg^−1^ and MTX: 10.0 mg kg^−1^) , and C/M@Alb NCs (CS: ≈100.0 mg kg^−1^ and MTX: 10.0 mg kg^−1^) at days 10 (day 0), 13 (day 3), and 16 (day 6) post-tumor tissue implantations. The tumor volumes and body weight were measured every third day during the next 18 d of post-treatment. The tumor volume was calculated by the formula (Eq. 4) previously detailed. Subsequently, the tumors harvested, weighted, and photographed. The freshly excised tumors and all major organs were washed with ice-cold 1× PBS (pH 7.4) and then fixed with 4% paraformaldehyde, embedded, sliced, and stained with H&E.

### Statistical analysis

Data were expressed as mean ± SD after triplicated independent experiments. The statistical significance was determined using Student’s *t* tests using GraphPad Prism 9 data processing software. Data with *P* value of >0.05 and <0.05 were stated as a statistically insignificant and significant, respectively.

## Results

### Preparation and characterization of C/M@Alb NCs

**Fig. 1.**
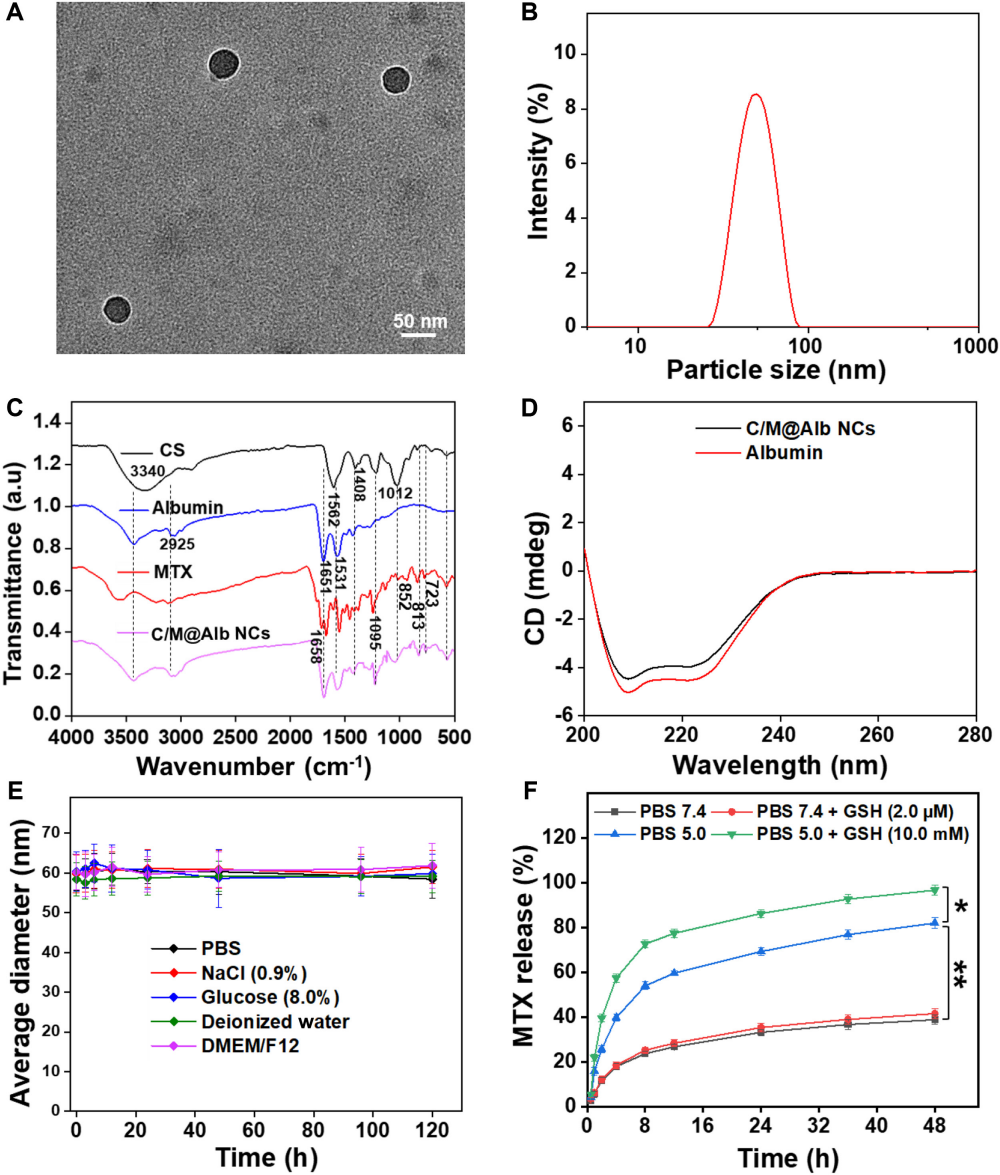
C/M@Alb NC physicochemical characterizations. (A) Representative TEM image of C/M@Alb NCs. (B) Hydrodynamic PS of C/M@Alb NCs. (C) FTIR spectrum of albumin, CS MTX, and C/M@Alb NCs. (D) Circular dichroism spectra of intact albumin and C/M@Alb NCs at concentration of 0.5 mg ml^−1^ of albumin. (E) Dispersion stability of C/M@Alb NCs at 4 °C in various dissolution media. (F) Accumulative percent release of MTX from C/M@Alb NCs in pH 7.4 and pH 5.0 with low (2.0 μM) and high (10.0 mM) amount of GSH (*n* = 3). Data are presented as a mean ± SD. **P < 0.01 and *P < 0.05 reveal significant differences calculated by Student’s *t* test.

C/M@Alb NCs were successfully prepared by albumin nanoreactor strategy under facile conditions by precipitating CS and MTX within hallow albumin nanocavity. The precipitation reaction between CS and MTX was triggered by the activation of CS with 4-(4,6-dimethoxy-1,3,5-triazin-2-yl)-4-methylmorpholinium chloride (DMT-MM) for 30 min and further allowed to react with MTX in the presence of albumin for 4 h at 37 °C. During the reaction, the amine group of MTX reacted with the carboxylic group (COOH) of preactivated CS within hallow albumin nanocavity followed by precipitation of MTX and CS as (MTX-NH-O-CS) inside the core, which directed the construction of C/M@Alb NCs. During incubation with preactivated CS, MTX-NH_2_ is transformed into positively charged MTX-NH^+^, which then combines with carbon ions (¯C=O) of CS and induces the nucleation and growth of MTX-NH-O-CS inside the hollow albumin nanocavity (Figs. S1 and S2). The successful fabrication of C/M@Alb NCs was initially confirmed by the round shape cage-like morphology having a mean diameter of 47.5 ± 5.2 nm observed under TEM (Fig. 1A). Besides, C/M@Alb NCs revealed a mean hydrodynamic PS of 59.5 ± 4.2 nm with a narrow and uniform size distribution [polydispersity index (PDI) 0.15] analyzed by Zetasizer (Fig. 1B). The slight increase in mean hydrodynamic PS of C/M@Alb NCs compared to mean diameter in dry form measured by TEM might be ascribed by the hydration layer around the albumin corona in aqueous solution. Hence, nanoformulation with 30- to 200-nm PS range is appropriate for enhanced tumor accumulation and cellular uptake [29]. C/M@Alb NCs showed surface ZP with an average value of −16.5 ± 2 mV (Fig. S3), as the negative surface charges are promising for the stability in physiological environment. Previously, it has been described that negative surface charged NPs are less toxic to the healthy cells as compared to positive charged NPs that harmed healthy cells and caused the aggregation of platelets and hemolysis of red blood cells (RBCs) [29].

C/M@Alb NCs showed satisfactory drug loading (8.5 ± 2.2% and 3.5 ± 1.8%) and EE (89.3 ± 3.6% and 65.8 ± 4.2%) in MTX and CS measured by an ultraviolet spectrometer and an ELISA kit, respectively.

The chemical interaction of MTX, CS, and albumin after C/M@Alb NCs was evaluated by FTIR spectroscopy. As revealed in Fig. 1C, the albumin FTIR spectrum revealed the attributed peaks at 1,531 cm^−1^ (–NH stretching and C–N vibrations), 1,651 cm^−1^ (C=O bending), and 2,925 cm^−1^ (–NH band) demonstrating amide III, II, and I peaks, respectively [30]. The FTIR spectrum of CS showed the representative peaks at 3,340 cm^−1^ (–OH bending), 1,408 and 1,612 cm^−1^ (–C=O bending), and 1,562 cm^−1^ (–NH amide II band) [31]. The FTIR spectrum of MTX showed corresponding peaks at ~3,000 to 3,400 cm^−1^ (carboxylic acid; OH group), 1,658 cm^−1^ (C=O vibration of carbonyl group), and 1,095 cm^−1^ (acyl; C–O stretching) [9]. As demonstrated in Fig. 1C, C/M@Alb NCs FTIR spectrum exhibited all the characteristic peaks of MTX, CS, and albumin, confirming the successful fabrication of C/M@Alb NCs comprising MTX, CS, and albumin with no visible structural changes during the fabrication process. Further, the crystalline MTX transformed into amorphous form with no visible sharp peak upon encapsulation into C/M@Alb NCs (Fig. S4). Such a transformation enhanced solubility of insoluble drug molecules [32]. Hence, high solubility rate drug molecule encourages higher drug absorption and permeation, which increases the therapeutic efficacy of drug [33,34]. Our results are coherent with previous report, where the conversion of crystalline lapatinib into amorphous form enhanced the solubility when encapsulated into poly(lactic-co-glycolic acid) (PLGA NPs) coated with albumin for tumor targeting [29].

Next, the secondary α-helical structure of albumin after C/M@Alb NCs preparation was investigated by circular dichroism (CD) spectroscopy. As shown in Fig. 1D, CD spectrum of C/M@Alb NCs displayed the attributed peaks at 222 and 208 nm corresponding to albumin α-helical structure, indicating that the preserved structure of albumin in C/M@Alb NCs is promising for in vivo tumor targeting.

The colloidal or dispersion stability is indispensable for the in vivo fate of nanomedicine. Upon entering to systemic circulation, nanomedicine interacts with diverse physiological conditions, which may cause dissociation or aggregation of nanomedicine. The colloidal or dispersion stability of C/M@Alb NCs was assessed in different dissolution medium including PBS (pH 7.4; 1×), NaCl solution (0.9%), glucose (8%), dH_2_O, and DMEM/F12 cell culture media. As shown in Fig. 1E, no considerable change was noticed in the mean hydrodynamic PS of C/M@Alb NCs for 5 d, indicating the admirable colloidal stability of C/M@Alb NCs in various dissolution media. The enhanced dispersion or colloidal stability of C/M@Alb NCs is due to the sufficient negative charges on the surface of albumin molecule that recruit strong repulsion between the individual NPs in solution form, which prevents the agglomeration or aggregation or of albumin NPs [29,35]. In addition, lyophilized C/M@Alb NCs exhibited an excellent accelerated stability at temperatures of −20 and 4 °C with no observable change in morphology, size, and drug content. However, at room temperature (25 °C), the drug content was stable, but morphology and PS show some changes. Surprisingly, high deterioration was observed with respect to drug content, PS, and morphology of C/M@Alb NCs stored at 37 °C (Table S1). Hence, the temperature (−20 and 4°C) indicates an optimal long-term storage condition for lyophilized C/M@Alb NCs.

### Dual pH/GSH-responsive MTX release from C/M@Alb NCs

The dual stimuli-responsive release profile of C/M@Alb NCs was assessed in PBS in response to pH and GSH (Fig. 1F). The results revealed that C/M@Alb NCs exhibited relatively slow at pH 7.4, mimicking the physiological pH and inadequate amount of MTX (38.76 ± 2.06%) during 48 h. Notably, C/M@Alb NCs exhibited increase release of MTX (81.92 ± 2.45%) at pH 5 (Fig. 1F) and the elevated release of MTX at pH 5.0 might be caused by the deformation of Alb structure and poor interaction among amino acids upon contact with acid environment [11,29]. Moreover, we studied the GSH-responsive drug release property of C/M@Alb NCs. MTX was rapidly released from C/M@Alb NCs in PBS (pH 5.0) containing high concentration of GSH (~10 mM mimicking tumor cell cytosol environment) as compared to MTX released from C/M@Alb NCs in PBS (pH 7.4) with very low GSH concentration (~2 μM mimicking extracellular matrix) within 48 h (Fig. 1F). Indeed, the total accumulative MTX release was about 96.56 ± 2.34% after 48 h in the release medium at pH 5.0, with high amount of GSH (10.0 mM). These results demonstrated the fast diffusion of encapsulated MTX in C/M@Alb NCs to the release medium (pH 5.0) with high amount of GSH (10.0 mM), as GSH caused the reduction of disulfide bonds (-S–S-) of albumin [36,37]. To further demonstrate the enzymatic degradation, C/M@Alb NCs were subjected to GSH treatment at pH 5.0 and 7.4, and then they were analyzed by sodium dodecyl sulfate–polyacrylamide gel electrophoresis (SDS-PAGE). C/M@Alb NCs possessed elevated degradation of albumin at higher GSH concentration (10 mM) compared to low GSH concentration (2.0 mM) (Fig. S5), owing to that C/M@Alb NCs with small PS are more accessible to a higher GSH concentration at the tumor site. Therefore, pH/GSH-responsive controllable drug release at the tumor site would maximize the inhibition of cancer cell growth and decreased the off-target systemic side effects of chemotherapy, which confirms the superiority of C/M@Alb NCs as a smart vehicle for cancer therapeutics.

The release profile of MTX from C/M@Alb NCs shown in Fig. 1F was fitted with the Peppas–Sahlin (Power law) mathematical model (Table S2), and the graphical depiction of the experimental data fitted with the mathematical model was demonstrated in Fig. S6. C/M@Alb NCs exhibited multifaceted drug release profiles, Fickian-type diffusion, and the cleavage of disulfide bonds (-S–S-) of albumin. According to the Peppas–Sahlin model, *n* value (0.54) <1 suggested Fickian diffusion. In addition, the Peppas–Sahlin coefficients suggested faster drug release with higher k1 value (28.11) and shifted toward polymer chain relaxation-based release mechanism with k2 more negative value (−2.06) in the presence of GSH, which causes the cleavage of disulfide bonds (-S–S-) of albumin.

### Cellular uptake, endocytic pathway, and intracellular distribution of C/M@Alb NCs

**Fig. 2.**
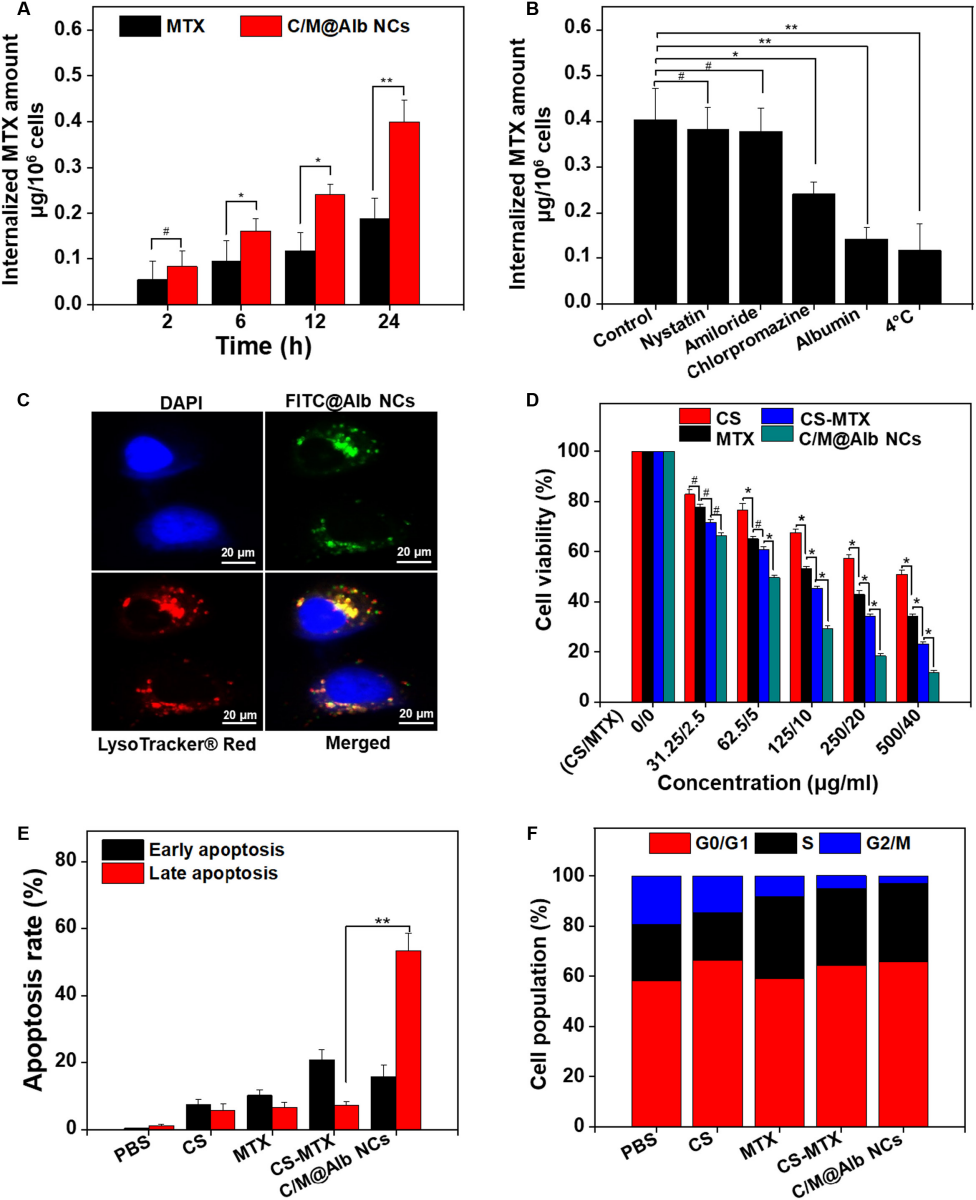
In vitro cellular uptake, endocytic pathway, and cytotoxicity studies. (A) Internalization of MTX in HT-29 cancer cells treated with MTX and C/M@Alb NCs (2.0 μg ml^−1^) for 2, 6, 12, and 24 h. (B) Endocytic pathway of C/M@Alb NCs in HT-29 cancer cells treated with various inhibitors (chlorpromazine, amiloride, nystatin, and albumin). (C) Intracellular distribution of FITC-tagged C/M@Alb NCs in HT-29 cancer cells stained with DAPI and LysoTracker Red. (D) Cell viability of HT-29 cancer cells treated with C/M@Alb NCs and free CS and MTX for 24 h. (E) Apoptotic level (%) of HT-29 cancer cells treated with C/M@Alb NCs and free CS and MTX for 24 h. (F) Cell cycle analysis of HT-29 cancer cells treated with C/M@Alb NCs and free CS and MTX for 24 h. Data are stated as a mean ± SD. *P < 0.05, **P < 0.01, and ^#^*P* > 0.05 show significant and insignificant differences, analyzed by Student’s *t* test.

To investigate the potential of C/M@Alb NCs to transport the cargo to intracellular environment, the cellular uptake of CS and MTX was assessed in HT-29 colon and E0771 breast cancer cells. The internalized concentration of MTX in cancer cells from C/M@Alb NCs demonstrated the significant enhancement with time dependency as compared to free MTX (Fig. 2A and Fig. S7). The maximum cellular uptake level was reached at 24 h of incubation [38]. In addition, the internalized amount of MTX in cancer cells from C/M@Alb NCs was found to be increased by increasing the concentration (0.5 to 2.0 μg ml^−1^) of MTX, incubated for 24 h, indicating concentration dependency (Fig. S8). Subsequently, the endocytic pathway of C/M@Alb NCs was demonstrated in HT-29 cells preincubated with different endocytic inhibitors. C/M@Alb NCs displayed a 39% decrease of MTX in the cellular uptake pretreatment with chlorpromazine, demonstrating a clathrin-mediated endocytic pathway of C/M@Alb NCs (Fig. 2B). Furthermore, preincubation with Alb at 4 °C also caused a substantial decrease in the cellular uptake of MTX (Fig. 2B), demonstrating an Alb receptor-mediated and energy-dependent cellular uptake of C/M@Alb NCs. Next, the intracellular distribution of FITC-tagged C/M@Alb NCs was assessed in HT-29 cancer cells by CLMS. C/M@Alb NCs exhibited high colocalization (85%) with lysosomes as a green fluorescence, which is promising for dual pH/GSH-responsive drug release at the tumor site.

### In vitro ROS scavenging ability and synergistic cytotoxicity

The ROS scavenging ability of C/M@Alb NCs in HT-29 cancer cells was estimated by dichlorodihydrofluorescein (DCFH) assay. During this assay, the esterase enzyme hydrolyzed H2DCF-DA, a nonfluorescent dye to DCFH, which afterward oxidized to DCF by intracellular ROS in the form of green fluorescence used as indicator for investigating the oxidative stress level inside the cells. The intracellular ROS scavenging ability in HT-29 cancer cells treated with C/M@Alb NCs and free CS and MTX was estimated. No visible green florescence was observed in HT-29 cancer cells treated with C/M@Alb NCs compared to free CS, MTX, and untreated cells (Fig. S9). Furthermore, C/M@Alb NCs and free CS exhibited an enhanced intracellular scavenging ability in a concentration-dependent way. However, C/M@Alb NCs at higher concentrations scavenged significantly higher ROS as compared to the free CS (Fig. S10). The enhanced antioxidant or ROS scavenging mediated by C/M@Alb NCs compared to free MTX and free CS might be caused by the improved cell uptake of C/M@Alb NCs via albumin receptors, which causes increased CS concentration inside the cells that subsequently caused enhanced ROS scavenging that inhibited the cell growth via oxidative stress, and triggered the cell death.

Next, the in vitro cytotoxic efficacy of C/M@Alb NCs against HT-29 and E0771 cells was estimated by CCK-8 assay. C/M@Alb NCs exhibited a dose-dependent anticancer efficacy with IC_50(MTX)_ 4.43 μg ml^−1^ (Fig. 2D), while the mixture of free CS-MTX had a relatively poor anticancer efficacy with IC_50(MTX)_ 7.65 μg ml^−1^, indicating that C/M@Alb NCs could act as a stimuli-responsive drug nanocarrier system to induce greater chemotherapeutic efficacy via enhanced cellular internalization and inherent acidity- and GSH-responsive drug release. In addition, C/M@Alb NCs further reduced the viability of HT-29 cells with IC_50(MTX)_ 3.63 μg ml^−1^, compared to the mixture of free CS-MTX with IC_50(MTX)_ 7.25 μg ml^−1^ at 48-h incubation (Fig. S11). Taken together, C/M@Alb NCs exhibited both dose- and time-dependent anticancer efficacy against HT-29 cancer cells. The higher cytotoxicity persuaded by C/M@Alb NCs could be attributed to the small PS of C/M@Alb NCs, stimuli-responsive drug release, and the albumin corona, which stimulate the enhanced cellular internalization of C/M@Alb NCs via albumin receptors. In addition, albumin-treated cells show high proliferation even at high concentration (500 μg/ml), indicating that albumin has no intrinsic cytotoxicity (Fig. S12). Besides, C/M@Alb NCs exhibited a strong anticancer efficacy against E0771 cells compared to HT-29 (Fig. S13). The strong anticancer efficacy of cells might be C/M@Alb NCs due the higher expression of SPARC on E0771 cells compared to HT-29 cells (Fig. S14). Moreover, the CI (0.606 < 1), indicating the synergistic effect between MTX and CS and in C/M@Alb NCs with an enhanced anticancer efficacy (Fig. S15).

### Cell apoptosis and cell cycle analysis

To investigate cytotoxicity mechanism of C/M@Alb NCs, flow cytometry (fluorescence-activated cell sorting) was employed to detect cell apoptosis level using annexin V–FITC/DAPI staining. As revealed in Fig. 2E and Fig. S16, HT-29 cells incubated with PBS have high viability with negligible apoptosis, whereas free MTX and CS caused a mild apoptosis with 6.1 ± 1.7% and 5.75 ± 1.5% early apoptosis and 10.2 ± 1.8% and 7.56 ± 1.7% late apoptosis, respectively. The mixture of free CS-MTX demonstrated more apoptotic level (20.8 ± 3.1%) at early stage, which is not desirable for cancer therapy due to severe off-target side effects [39]. Importantly, C/M@Alb NCs causes higher cell apoptosis of 53.3 ± 5.3% at late stage via synergistic effect between CS and MTX, indicating a significantly higher anticancer efficacy against HT-29 cancer cells due to an enhanced cellular internalization of C/M@Alb NCs via albumin receptors (Fig. 2E and Fig. S16).

Cell growth depends on the cell cycle process. Hence, we investigated the effect of C/M@Alb NCs on the cell cycle process of HT-29 cancer cells. As depicted in Fig. 2F and Fig. S17, free CS treatment arrests the G_0_/G_1_ phase of the cell cycle in HT-29 cancer cells and stops them from entering the S phase of the cell cycle [5]. The G phase is promising for fast metabolism, enhances the protein and RNA synthesis, and subsequently increases cell growth. Furthermore, the G_1_ phase also provides energy for replication of DNA in the S phase [40]. Thus, the delay in the G_0_/G_1_ phase could efficiently avert the cells from entering the S phase, delaying the synthesis of DNA and reducing cell growth. Free MTX arrests the S phase of the cell cycle and prevents the cells from entering the G_2_/M phase [41]. The mixture of CS-MTX and C/M@Alb NCs arrested the cell cycle at the G_0_/G_1_ phase and S phase, thus stopping the cells from entering the G_2_/M phase of the cell cycle. Importantly, C/M@Alb NCs distinctly decreased the population of cells in the G_2_/M phase compared to mixture-free CS-MTX. In general, the results revealed that C/M@Alb NCs arrest the cell cycle of HT-29 cancer cells at G_0_/G_1_ and S phases and thus inhibit cell growth cooperatively.

### Blood residence time and biodistribution of C/M@Alb NCs

**Fig. 3.**
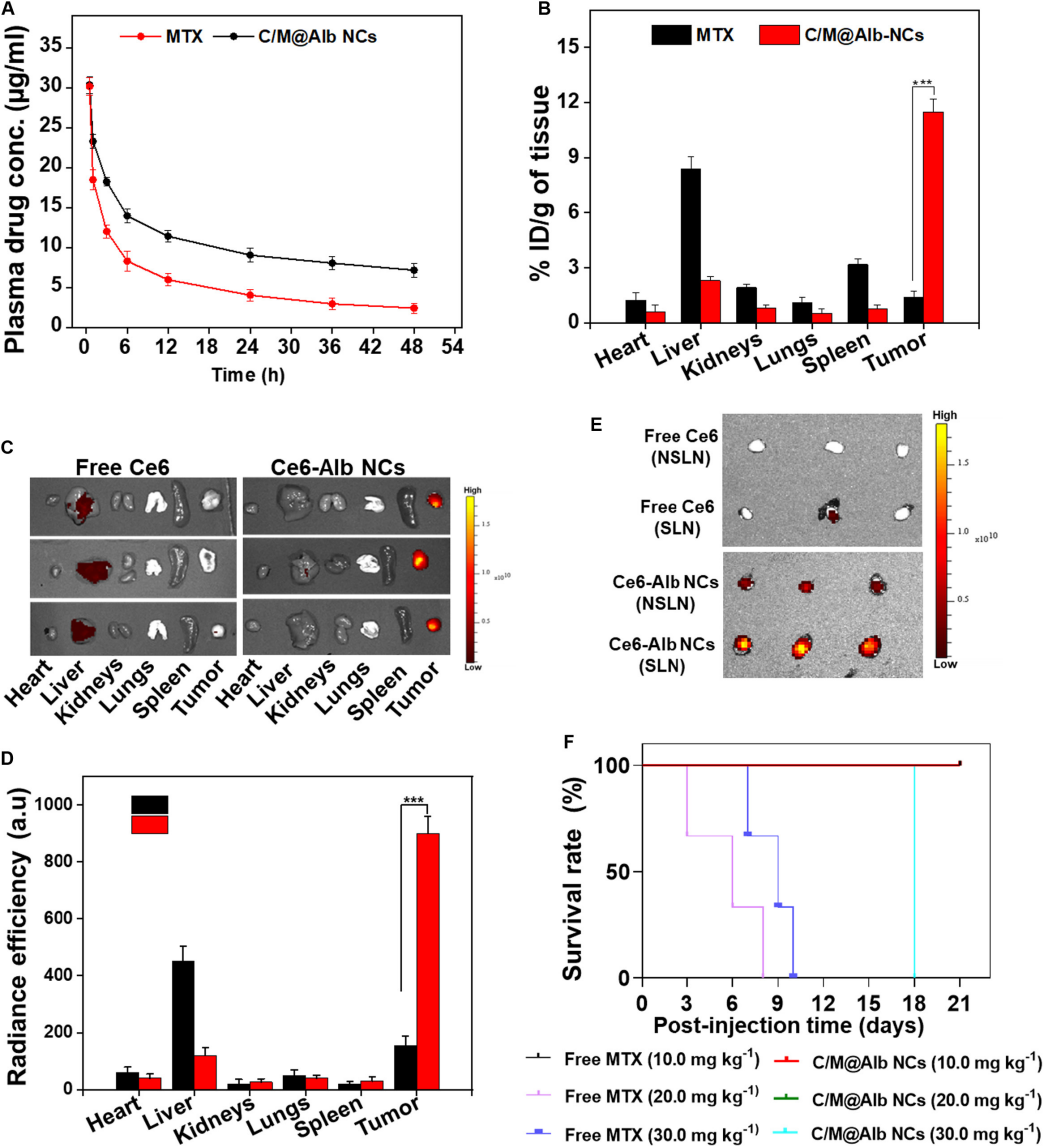
(A) Pharmacokinetic profiles of C/M@Alb NCs. (B) Distribution of C/M@Alb NCs to tumors and major organs. (C) NIRF ex vivo images of harvested HT-29 tumors and major organs after 24 h of injection. (D) Quantification of radiance efficiency from (C). (E) NIRF ex vivo images of harvested LNs after 24 h of injection. (F) Demonstrative survival curve of the C57BL/6 mice injected with C/M@Alb NCs and free MTX doses (10.0, 20.0, and 30.0 mg kg^−1^). Data are itemized as mean ± SD, and ***P < 0.001and *P < 0.05 specify statistically significant difference analyzed by Student’s *t* test.

The blood residence time of C/M@Alb NCs was determined by pharmacokinetic profile assessment after injecting a single dose of C/M@Alb NCs (10.0 mg kg^−1^ of MTX) into ICR BALB/c male healthy mice. C/M@Alb NCs exhibited MTX elimination half-lives (*t*_1/2β_) of 5.78 h, which is 4 times longer than free MTX (1.15 h), suggesting prolonged blood circulation time of C/M@Alb NCs. Indeed, free MTX was rapidly eliminated from the blood circulation as shown by the dramatic decline in the plasma MTX concentration after 1.5 h of injection (Fig. 3A). Furthermore, the AUC_0~∞_ of C/M@Alb NCs was determined to be 90.5, which is 4-fold higher than free MTX (Table S3).

In addition, the blood residence time of C/M@Alb NCs was validated by ex vivo fluorescence imaging of the blood samples collected from the mice after a single-dose (3.5 mg kg^−1^) injection of Ce6-conjugated C/M@Alb NCs and free Ce6 (Fig. S18). Ce6-conjugated C/M@Alb NCs exhibited prolonged blood residence time and remained in blood circulation with strong fluorescence signal for longer time compared to free Ce6, which was quickly eliminated from the bloodstream with no noticeable fluorescence signal after 3 h of injection. The enhanced blood residence time of C/M@Alb NCs may be attributed to prolonged half-life of Alb in the blood and the small PS (≈ 60 nm), likely empowering the in vivo targeted delivery to the tumor site [42].

Next, the tumor targeting potential of C/M@Alb NCs was evaluated by measuring the distribution of MTX from C/M@Alb NCs to the tumor and major organs (liver, heart, lungs, spleen, and kidney) after 24 h of single injection at an MTX dose of 10.0 mg kg^−1^. C/M@Alb NCs resulted in higher tumor accumulations of 11.47 injected dose (ID) % g^−1^ of MTX, which signifies 8.3-fold increase compared to free MTX (Fig. 3B). Furthermore, C/M@Alb NCs decrease the distribution of MTX to major organs, demonstrating the target-specific transport of MTX to the tumor milieu via Alb nanocarrier. The tumor targeting ability of C/M@Alb NCs was also validated by ex vivo near-infrared fluorescence (NIRF) imaging of the excised tumors and major organs, after a single-dose (3.5 mg kg^−1^) injection of Ce6-conjugated C/M@Alb NCs into tumor-bearing mice. Ce6-conjugated C/M@Alb NCs exhibited stronger fluorescence signals with 4-fold increase as compared to free Ce6, indicating improved tumor accumulation via albumin receptors (Fig. 3C and D). The prolonged blood residence time of C/M@Alb NCs and permeation through tumor leaky vasculature known as an enhanced permeability and retention (EPR) effect could be one of the probable reasons for greater tumor accumulation, but targeting tumor via EPR effect depends on tumor nature. In additiion, rapidly proliferating cancer cells use Alb as a food and energy source; therefore, C/M@Alb NCs could be readily internalized by the aggressive cancer cells to fulfil their energy requirements for survival [43]. On the other side, the inferior distribution of C/M@Alb NCs to major healthy organs is maybe due to the recycling mechanism of albumin via neonatal Fc receptor (FcRn) [44]. Moreover, the extravasation of C/M@Alb NCs to the SLN and NSLN was evaluated. The Ce6-conjugated C/M@Alb NCs displayed distinctly higher extravasation to SLN and NSLN, and the enhanced accumulation in the excised SLN around the tumors would be promising for tumor metastasis inhibition as SLN is considered as a first source for cancer metastasis [27].

Prior to exploring the in vivo anticancer efficac*y*, the maximum tolerated dose (MTD) of C/M@Alb NCs was estimated by injecting the escalating doses of C/M@Alb NCs and free MTX in healthy C57/BL6 mice, following the protocol from Liu et al. [45]. The mice injected with C/M@Alb NCs (10.0, 20.0, and 30.0 mg kg^−1^ MTX) exhibited body weight (BW) loss initially during the first week after injection and recovered to the initial BW with negligible morbidity (one mouse died at day 18 injected with C/M@Alb NCs dose 30 mg kg^−1^ MTX) during the experimental period (Fig. 3F and Fig. S19). However, the mice received higher doses of free MTX (20.0 and 30.0 mg kg^−1^ MTX), displayed symptoms such as severe BW loss, laziness, and hair standing, and subsequently died during the first 2 weeks of post-injection, demonstrating the severe toxicity at higher doses of free MTX. Furthermore, the mice that received 10.0 mg kg^−1^ of free MTX survived with 20% BW loss compared to initial BW. Therefore, according to overall survival rate and body weight, the dose of C/M@Alb NCs tolerated by the C57/BL6 mice is around 30.0 mg kg^−1^, 3-fold higher as compared to MTD of free MTX (10.0 mg kg^−1^). Furthermore, the different concentrations of C/M@Alb NCs (with respect to MTX) exhibited low hemolysis (hemolysis rate < 2%) to RBCs when incubated for 1 h at 37 °C (Fig. S20). Thus, these findings further provide evidence of the biocompatibility and biosafety of C/M@Alb NCs for potential in vivo application.

### Inhibition of HT-29 subcutaneous tumors

**Fig. 4.**
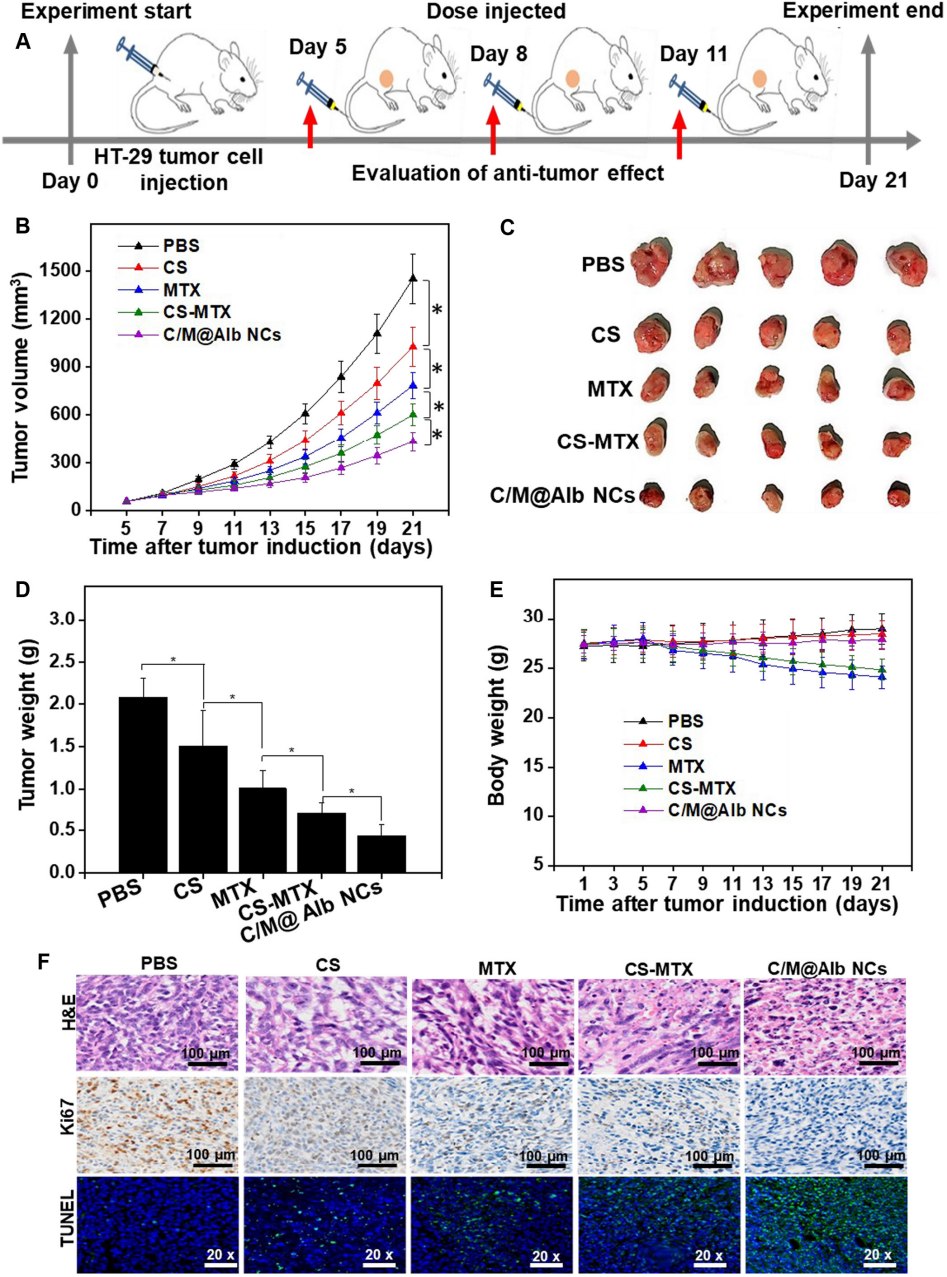
In vivo tumor repressive efficacy of C/M@Alb NCs against HT-29 subcutaneous tumors. (A) Illustration of tumor initiation and treatment regimen of the HT-29 subcutaneous tumor model. (B) Growth profile of HT-29 subcutaneous primary tumors. (C) Photographs of the harvested HT-29 subcutaneous tumors after treatment. (D) Average weight of the harvested HT-29 subcutaneous tumors. (E) Average body weight of tumor-bearing nude mice. (F) H&E-, Ki67-, and TUNEL-stained images of the HT-29 subcutaneous tumors after treatment. Data stated as an average value ± SD (*n* = 5). *P < 0.05 shows a statistically significant difference.

The synergistic in vivo tumor-suppressive efficacy of C/M@Alb NCs was evaluated against HT-29 subcutaneous tumors using 3 doses of injections, and the tumor volume was monitored for 21 d (Fig. 4A). As described in Fig. 4B, the volume of tumors of mice treated with PBS quickly increased and attained an average volume (1,452.72 ± 157.34 mm^3^) of tumor at day 21 of post-tumor initiation. Free MTX and CS expressively repressed the growth of tumors as compared to the PBS group (Fig. 4B). Furthermore, the mixture of free MTX and CS exhibited stronger antitumor efficacy and suppressed the tumor growth more compared to free MTX and CS when injected alone, demonstrating the synergism between MTX and CS. However, free MTX, free CS, and the mixture of free CS-MTX suppressed the growth of tumor initially during the first few days of treatment and the tumors gain the pace of growth after the third injection, indicating the fast elimination of free drugs from systemic circulation. Fascinatingly, C/M@Alb NCs significantly suppressed the tumor growth via targeted synergy when compared with free MTX, free CS, and the mixture of free CS-MTX. Afterward, the excised tumors at day 21 were visually observed and the tumors of mice treated with C/M@Alb NCs exhibited lower weight (Fig. 4C and D). The enhanced tumor-suppressive efficacy of C/M@Alb NCs might be facilitated by the small PS, prolonged systemic circulation, and albumin receptor-based tumor accumulation of C/M@Alb NCs. Besides, the prolonged half-life of albumin also promises the extended retention of C/M@Alb NCs in the systemic circulation, which favors the tumor extravasation via EPR effect. Notably, the C/M@Alb NC-treated mice sustained a healthy BW during the treatment period (Fig. 4E), indicating the biosafety of C/M@Alb NCs.

Next, the enhanced tumor-suppressive efficacy of C/M@Alb NCs was validated by H&E, Ki67, and TUNEL (terminal deoxynucleotidyl transferase–mediated deoxyuridine triphosphate nick end labeling) staining following previous protocols with some modifications [46]. H&E-stained tumor section suggests that C/M@Alb NCs caused a most severe cell damage with intense hemorrhagic inflammation at the tumor due to the strong chemotherapeutic effect (Fig. 4F), while PBS-treated tumors as control displayed no obvious tumor damage and showed well-organized tumor architecture. Concordantly, images of Ki67-stained tumor section indicated that C/M@Alb NCs exhibited the reduced number of Ki67-positive cells, revealing a considerable chemotherapeutic synergy of MTX and CS in C/M@Alb NCs for inhibition of tumor growth (Fig. 4F). Furthermore, TUNEL-stained tumor section treated with C/M@Alb NCs revealed more strong green signals, indicating severe cell death in the tumors (Fig. 4F).

### Inhibition of E0771 orthotopic tumors and lung metastasis

**Fig. 5.**
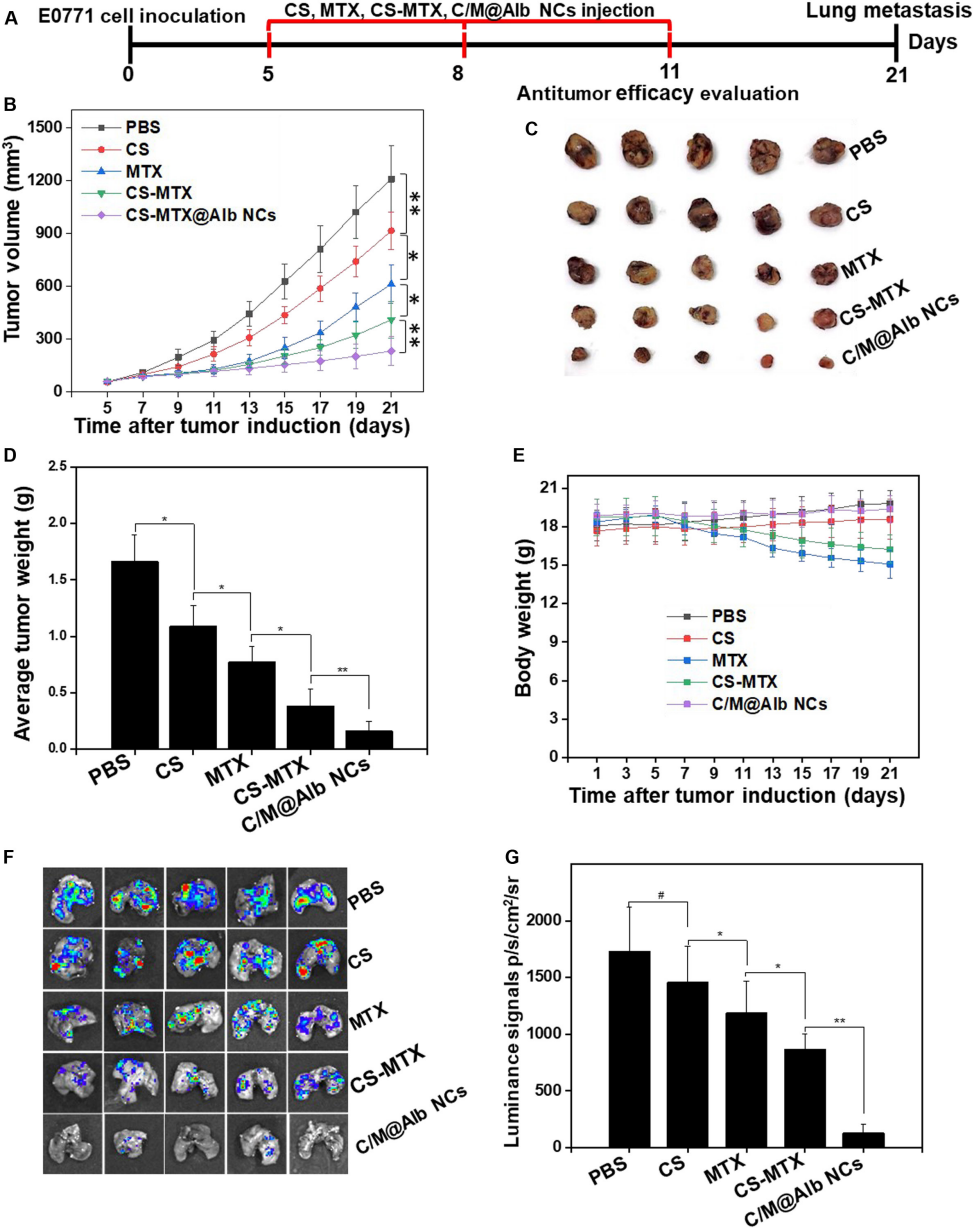
In vivo tumor-suppressive efficacy of C/M@Alb NCs against E0771 orthotopic breast tumors induced in C57BL/6 mice. (A) Illustration of tumor initiation and treatment regimen of the E0771 orthotopic breast tumor model. (B) Tumor growth profile of E0771 orthotopic breast tumors. (C) Photographs of the E0771 orthotopic breast tumors excised at day 21. (D) Average weight of the excised E0771 orthotopic breast tumors after treatment. (E) Average body weight of E0771 breast tumor-bearing C57BL/6 mice in the experimental period (21 d). (F) Bioluminescence images of the excised lungs from E0771 breast tumor-bearing C57BL/6 mice. (G) Quantification of bioluminescence in lungs. Data expressed as an average value ± SD (*n* = 5). **P < 0.01 and *P < 0.05 display significant differences.

The in vivo tumor-suppressive efficacy of C/M@Alb NCs was evaluated against highly aggressive and metastatic orthotopic E0771 murine breast tumor, which invaded the lung tissue habitually with high mortality rate. C/M@Alb NCs (10.0 mg kg^−1^ MTX and 100.0 mg kg^−1^ CS) were intravenously injected to the orthotopic E0771 tumor-bearing C57BL/6, and the tumor volume was measured for 21 d (Fig. 5A). Free MTX, free CS, and the mixture of free CS and MTX significantly repressed the growth of tumors; however, the tumor continues its growth after the third injection, which is mainly due to the rapid clearance of free drugs from blood circulation. Notably, C/M@Alb NCs significantly inhibited the growth of tumors as compared to free MTX, free CS, and the mixture of free CS-MTX via targeted synergy (Fig. 5B). In addition, smallest tumors were noticed for C/M@Alb NC treatment after visually observing the harvested tumors at day 21 (Fig. 5C and D). Moreover, C/M@Alb NCs sustained a healthy BW during the treatment period (Fig. 4E), confirming the C/M@Alb NC biosafety without any side effects (Fig. 5E). The admirable anticancer efficacy of C/M@Alb NCs validating dual pH/GSH-responsive drug release prolonged blood residence time and higher tumor accumulation.

Afterward, the anti-metastasis ability of C/M@Alb NCs was also evaluated by measuring bioluminescence in lung harvested from orthotopic E0771 breast tumor-bearing C57/BL6 mice at day 21. Notably, a negligible bioluminescence was observed in lungs harvested from C/M@Alb NC-treated mice, whereas lungs harvested from PBS, free MTX, free CS, and free CS-MTX treated mice exhibited stronger bioluminescence (Fig. 5F and G). This suggests that C/M@Alb NCs significantly inhibit the invasion and metastasis of cancer cells from primary site (breast pad) to secondary site (lungs). The enhanced tumor-suppressive efficacy of C/M@Alb NCs was confirmed by H&E, Ki67, and TUNEL staining of the E0771 primary tumor section, which revealed that C/M@Alb NCs caused severe damages to tumor cell with stronger apoptosis (Fig. S21), confirming the cooperative potent antitumor efficiency of C/M@Alb NCs against primary E0771 breast tumors and lung metastasis inhibition.

### Inhibition of patient-derived lung tumors

**Fig. 6.**
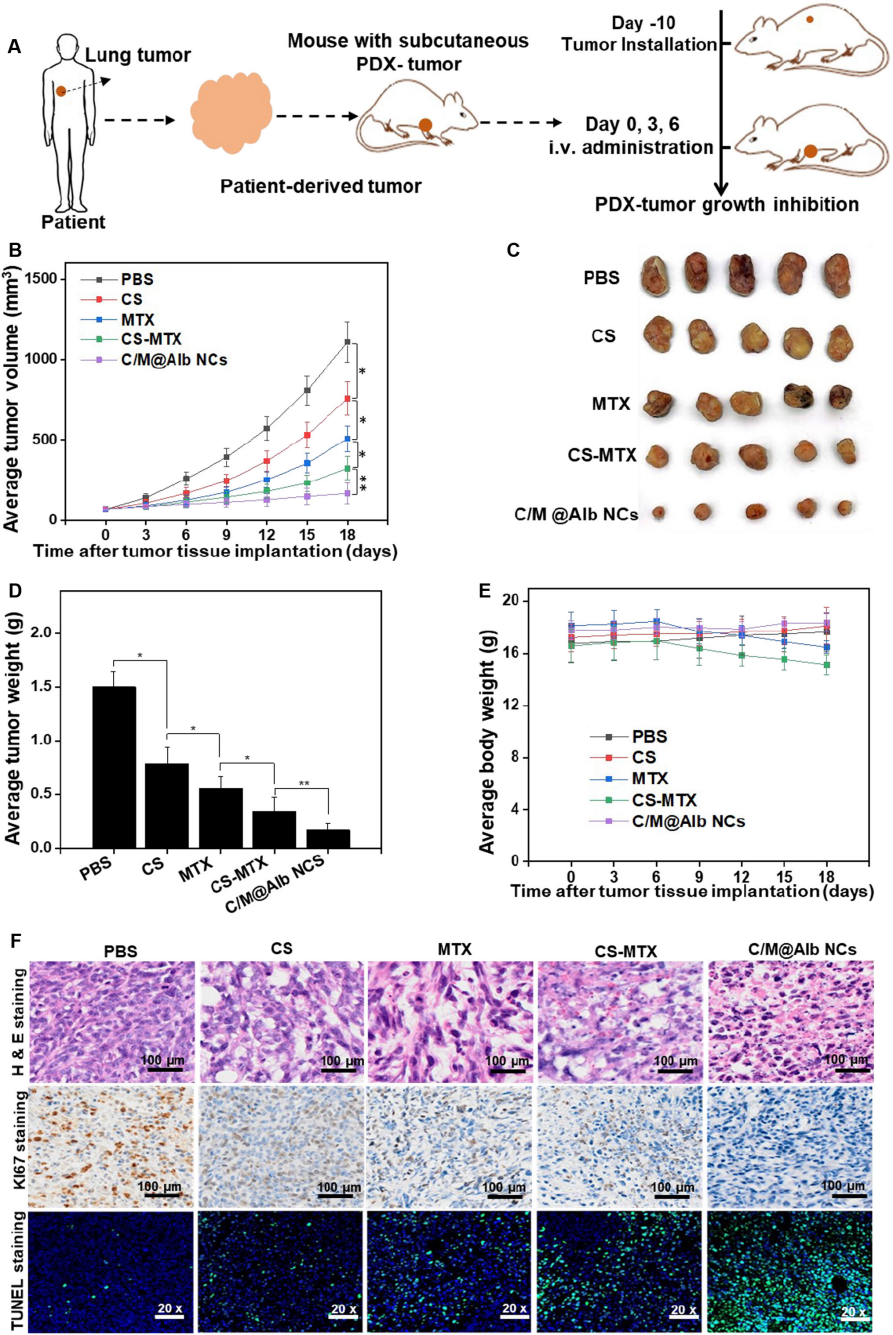
In vivo anticancer efficacy of C/M@Alb NCs against PDX primary tumors. (A) PDX tumor inoculation and dosing schedule. (B) Growth profiles of PDX tumors after receiving treatment. (C) Photographs of the harvested PDX tumors. (D) Average weight of the harvested PDX tumors. (E) Average body weight of PDX tumor-bearing nude mice throughout the experimental period. (F) H&E-, Ki67-, and TUNEL-stained images of PDX primary tumors. Data stated as an average value ± SD (*n* = 5). *P < 0.05 and **P < 0.01 display the significant difference.

Inspired by the tumor-suppressive efficacy of C/M@Alb NCs against orthotopic breast cancer and lung metastasis, we further exploited the therapeutic efficacy of C/M@Alb NCs against hostile PDX lung tumors. For this, PDX tumors were successfully inoculated in immunodeficient/nude mice followed by treatment of 3 doses of injection (Fig. 6A). The growth profile of PDX tumors was monitored during experimental period (18 d). The tumors of the PBS-treated mice aggressively grow compared to free CS, MTX, and the mixture of CS-MTX (Fig. 6B). Notably, the growth of tumors were significantly delayed in mice treated with free CS and free MTX compared to the PBS group (*P* < 0.05). However, the mixture of free CS-MTX exhibited more tumor-suppressive efficacy compared to free MTX and free CS. Thus, the higher tumor inhibition efficacy is more probably due to the synergism between MTX and CS as a combinatorial therapy. The free drugs caused temporary retardation of tumor growth during treatment, and apparent tumor regrowth occurred after the third injection (day 9). Predominantly, C/M@Alb NCs exhibited a significantly higher tumor inhibition efficacy and no apparent tumor growth relapse was perceived during the experimental period of 18 d (Fig. 6C). Moreover, the harvested tumors from C/M@Alb NC-treated mice exhibited noticeably lower tumor weight (Fig. 6C and D). In addition, C/M@Alb NC-treated mice maintained their BW, demonstrating enhanced biosafety of C/M@Alb NCs (Fig. 6E). Further, the H&E-, Ki67-, and TUNEL-stained images of PDX lung tumors revealed that C/M@Alb NCs caused severe damages to tumor tissue with retard tumor growth and enhanced cell apoptosis (Fig. 6F), validating the potent anticancer efficacy of C/M@Alb NCs for PDX primary tumor treatment.

### Long-term systemic toxicity of C/M@Alb NCs

**Fig. 7.**
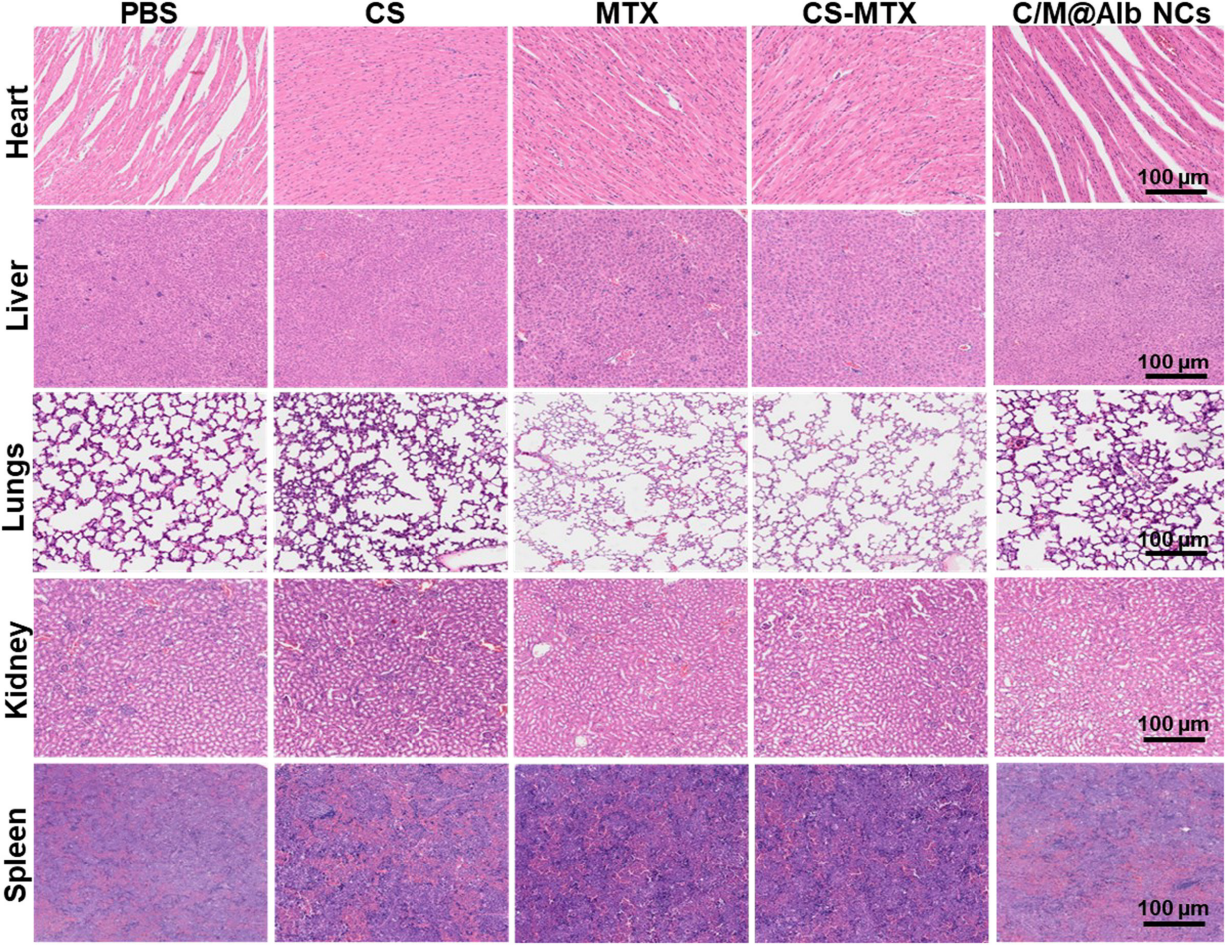
Histological examination (scale bar, 100 μm) of major organs after treatment with CS, MTX, CS-MTX, and C/M@Alb NCs at the CS and MTX dose of ≈100 and 10 mg kg^−1^, respectively.

The long-term in vivo safety is critical for determining the clinical fate of nanomedicine. Therefore, C/M@Alb NCs were injected to the tumor-bearing mice (*n* = 5 per group) at the same doses of the tumor inhibition for the systemic toxicity evaluation. The H&E-stained images of the major organs, such as liver, heart, spleen, lungs, and kidney excised from mice injected with C/M@Alb NCs, show no apparent toxicity, indicating enhanced systemic long-term safety of C/M@Alb NCs (Fig. 7).

Besides, the biosafety of C/M@Alb NCs was also evaluated by serum biochemical analyses after injection (day 21). As denoted in Fig. S22, the liver function and kidney function markers such as alanine aminotransferase (ALT), aspartate aminotransferase (AST), alkaline phosphatase (ALP), blood urea nitrogen (BUN), and serum creatinine (CRE) shows value in normal range, suggesting that C/M@Alb NCs have no evident toxic effects on kidneys and on liver functions, demonstrating the admirable systemic long-term biosafety of C/M@Alb NCs. In addition, no obvious change was observed in the pro-inflammatory cytokine interleukin-6 (IL-6), IL-1β, and tumor necrosis factor-α (TNF-α) levels in serum obtained from mice treated with C/M@Alb NCs when compared to untreated healthy mice (Fig. S23). However, the cytokine level drastically increased in C/M@ovalbumin (OvAlb) NC-treated mouse serum as ovalbumin is known for its immunogenic response. In short, C/M@Alb NCs with negligible hemolysis rate and non-immunogenicity confirmed an excellent biosafety C/M@Alb NCs for in vivo applications.

## Conclusion

This work employed albumin nanoreactor strategy to develop albumin nanocages with MTX and CS with protein corona and suitable PS for effective tumor- and LN-targeted cancer therapy and lung metastasis inhibition. MTX was precisely precipitated with preactivated CS within albumin nanocages via confined precipitation reaction. As a result, C/M@Alb NCs with average PS of 59.5 ± 4.2 nm was fabricated by precipitating MTX and CS as MTX-NH-O-CS within the hollow albumin nanocavity. The as-prepared C/M@Alb NCs possess superior characteristics such as suitable PS, appropriate drug loading, first-rate colloidal stability, and dual pH/GSH-responsive controlled drug release. Importantly, C/M@Alb NCs exhibited enhanced and albumin receptor-mediated cellular uptake, intracellular ROS scavenging ability, extended blood residence time, and favorable tumor accumulation and LN extravasation, finally leading to the potent antitumor efficacy against HT-29 subcutaneous colon tumors, E0771 orthotopic breast tumors, as well as PDX primary lung tumors with reduced systemic toxicity, providing a clinically satisfactory strategy for enhancing the therapeutic efficacy and clinical translation.

## Ethical Approval

All mice experiments were checked and approved by the research ethics committee of Hangzhou Institute of Medicine, Chinese Academy of Science (CAS), Hangzhou, China (approval no.: 2023R0027). The 3R rule was respected.

## Data Availability

All data produced during the study are presented in the manuscript and the Supplementary Materials.
