# Supplementary material for: Albumin Nanocages with Methotrexate and Chondroitin Sulfate as a Dual pH/GSH-Responsive Tumor Targeting Nanomedicine for Synergistic Cancer Therapy
Source: Biomater Res. 2025 Sep 3;29:0245. doi: 10.34133/bmr.0245 (PMC12407586; doi:10.34133/bmr.0245)
Supplement: Supplementary 1 — Materials and Methods Figs. S1 to S23 Tables S1 to S3 [file bmr.0245.f0245.docx]

**Supplemental Materials**

**Albumin Nanocages with Methotrexate and Chondroitin Sulfate as a Dual pH/GSH-responsive Tumor Targeting Nanomedicine for Synergistic Cancer Therapy**

Haroon Iqbal^1, 2^, Anam Razzaq^3^, Ziyin Yuan^1^, Lina Zhai^1^, Yue Wang^1^, Uzair Ur-Rehman^1^, Lv Man^1^, Jun Xin^1^_,_ Xin Ning^1^, Yuanbo Liang^2^*, Run Xiao^1,2^*

^1^ Zhejiang Cancer Hospital, Hangzhou Institute of Medicine, Chinese Academy of Sciences, Hangzhou, Zhejiang 310022, China.

^2^ *Eye Research Center, Hangzhou Institute of Medicine, Chinese Academy of Sciences, Eye Hospital, Wenzhou Medical University, Hangzhou, 310018, China*

^3^ Jiangsu Key Laboratory of Neuropsychiatric Diseases, College of Pharmaceutical Science, Soochow University, Suzhou, 215123, China

*Correspondence authors:

*Eye Research Center, Hangzhou Institute of Medicine, Chinese Academy of Sciences, Eye Hospital, Wenzhou Medical University, Hangzhou, 310018, China.*

Zhejiang Cancer Hospital, Hangzhou Institute of Medicine, Chinese Academy of Sciences, Hangzhou, Zhejiang 310022, China.

Email: L.Y.B: [yuanobliang@wmu.edu.cn](mailto:yuanobliang@wmu.edu.cn) ; X.R: [xiaorun1984@ucas.ac.cn](mailto:xiaorun1984@ucas.ac.cn)

Figure S1-S23

Table S1-S3

**Materials and methods**

**Intracellular Free Radical Scavenging**

To determine the intracellular ROS scavenging capacity of C/M@Alb NCs, dichlorofluorescein (DCFH) assay was performed in HT-29 cells. Briefly, HT-29 cells (1×10^5^ cells/well) were seeded in glass-bottom dishes followed by overnight incubation and exposed to hydrogen peroxide (H_2_O_2_) for next 12 h. Subsequently, the cells were carefully washed after removing the medium and then supplied fresh medium containing CS, MTX, C/M@Alb NCs (10.0 μg/mL CS, 5.0 μg/mL MTX) and further incubated for 12 h. Afterwards, the treated cells were washed thrice with 1x PBS (pH 7.4) and stained with DAPI (5.0 μg/mL) for 5 minutes followed by three-time washing with 1x PBS (pH 7.4) and DCFH-DA (10 µM) staining for 30 min in the dark. Afterward, green fluorescence signals were detected inside the cells by confocal laser scanning microscope (CLSM).

**Blood biocompatibility assay**

To evaluate the biocompatibility of C/M@Alb NCs, fresh blood was taken from the mouse eye-socket vein using capillary tube and placed in heparinized tubes. The blood was centrifuged at 5000 rpm for 3 min at 4 ºC to obtained red blood cells (RBCs) and washed the RBCs with ice-cold PBS five time to remove plasma. Next, 2% RBCs suspension (v/v) in PBS incubated with C/M@Alb NCs for 1 h at 37 ℃. Milli-Q water and PBS were used as positive and negative controls, respectively. After incubation, the suspension was centrifuged at 5000 rpm for 3 min, and the supernatant (100 μL) was added to each well of transparent 96-well plate followed by measuring the absorbance at 545 nm multimode microplate reader (Spark®, Tecan AG, Switzerland). Finally, hemolysis rate (HR) was determined by the following equation:

$HR (\%) =\frac{ANCs-ANC}{ANC-APC}$

Whereas A*_NCs_*: absorbance of C/M@Alb NCs, *A_NC_*: absorbance of negative control, *A_PC_*: absorbance of positive control.

**Conjugation of Ce6 with C/M@Alb NCs**

The carboxylic acid group of Ce6 was conjugated with free amine group in albumin through carbodiimide catalyzed amide formation, in which EDC (4.8 mg) and NHS (4.45 mg) were added to 2.0 mL Ce6 solution (3.75 mg mL^-1^) for 1 h. Then, this mixture was added to 5.0 mL solution of C/M@Alb NCs and stirred in the dark for 12 h. Finally, Ce6-conjugated C/M@Alb NCs were obtained after the centrifuge ultrafiltration (10k MW, 2000 rpm, 10 min/each) in distilled water.

**TUNEL Assay and KI67 Staining**

For TUNEL assay, the excised tumor sections were incubated with proteinase K for antigenic determinant retrieval followed by treatment with Triton X-100 (0.1%) solution for enhanced cell permeability. The tumor sections were then sited on glass-slide and sealed with a coverslip after TUNEL according to the manufacturer’s protocol. The images were obtained by using CLSM. For Ki67 staining, the paraffinized tumor sections were dehydrated, followed by EDTA treatment for epitope retrieval. Tumor sections were then exposed to hydrogen peroxide (H_2_O_2_) solution (3%) for endogenous peroxidase blocking, and before the tumor sections were treated with anti-Ki-67 primary antibody at 4 °C for 12 h, and incubated secondary antibodies for 1 h. After careful washing, the tumor sections were incubated with diaminobenzidine solution to generate brown color as positive signals. Hematoxylin was used to stain nuclei. In the end, images of the stained slices were captured by optical microscopy.

**Long-term Toxicity Evaluation**

After treatment period (day 21), the major organs including heart, liver, kidney, spleen and lungs from the nude BALB/c mice were excised, sectioned, sited on glass-slide, stained with H&E and sealed with a coverslip. The H&E-stained slides of major organs were photographed by light microscopy to evaluate organ specific long-term toxicity.

***In vivo* Toxicity Evaluation**

For *in vivo* toxicity evaluation, *~* 0.5 mL blood sample was collected from retro-orbital vein of each mouse treated with CS, MTX, CS-MTX and C/M@Alb NCs. The PBS-treated mice were used as control. Blood samples were cold-centrifuged (4 ºC) at 5000 rpm for 15 min to collect the serum. Then, the serum (*~*200 μL) of each sample was analyzed for blood biochemistry using the serum automatic analysis machine*.* In addition, we evaluated the impact of C/M@Alb NCs to the immune system in comparison to C/M@OvAlb NCs after single iv injection. Briefly, C57/BL6 mice (n=5) was injected with C/M@Alb NCs and C/M@OvAlb NPs (≈100 mg/kg CS and 10 mg/Kg MTX). Untreated mice (n=5) were used as control group. On day 7 of injection, *~* 0.5 mL blood sample was collected from retro-orbital vein of each mouse into heparinized tubes and obtained the serum after centrifugation. The serum was analyzed for cytokines such as interleukin-6 (IL-6), interleukin-1 beta (IL-1β) and tissue necrosis factor alpha (TNF-α) level by ELISA kits.

**
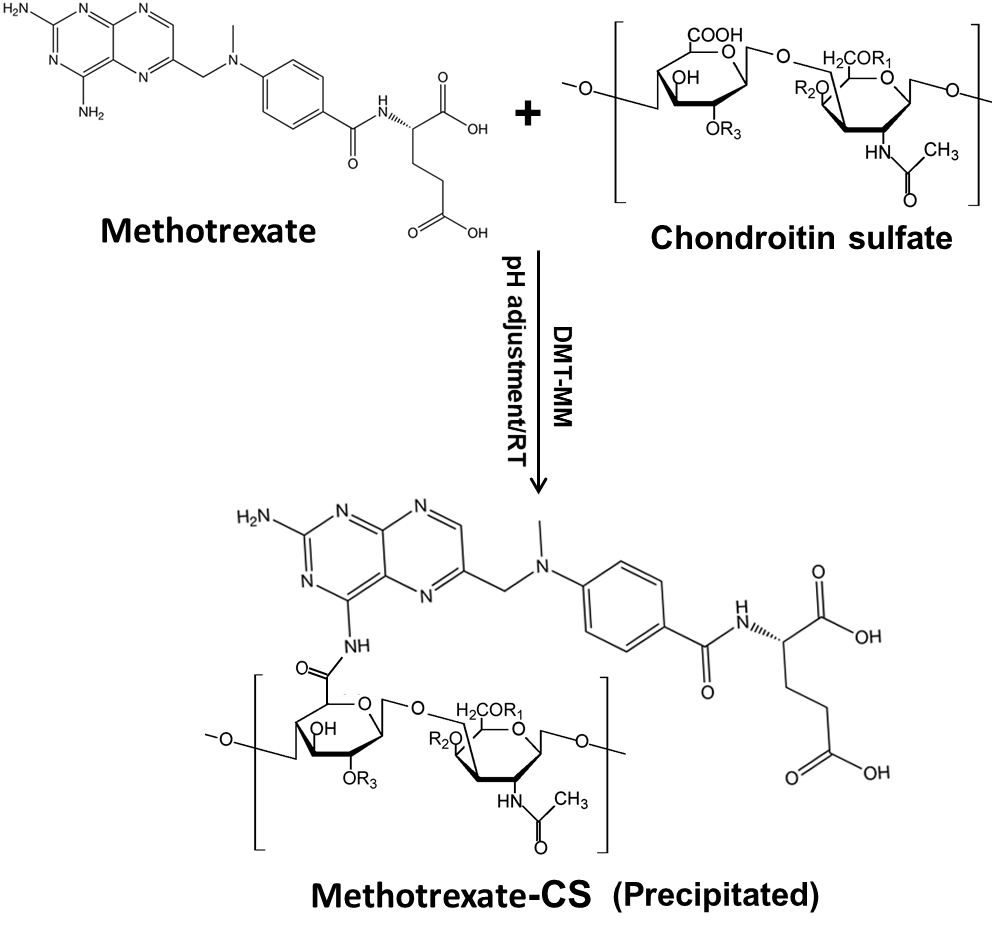
** **Figure S1.** The reaction between chondroitin sulfate and methotrexate within albumin hallow nanocages. R1=H, R2=SO_3_^–^ and R=3.


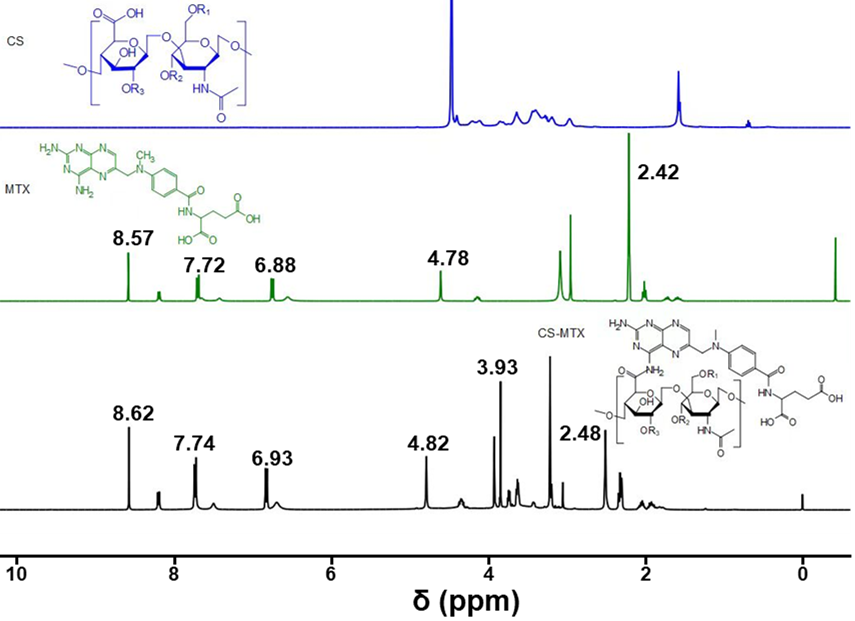


**Figure S2.** The NMR spectra of CS, MTX, and MTX-CS bioconjugates. The peaks at 6.88 (2H, d, J= 10.1 Hz) and 7.72 (2H, d, J=10.1 Hz) can be assigned to the benzoyl group of MTX. The peaks at 4.78 (2H, s) can be assigned to the methylene next to the 2, 4-diamino-6 pteridinyl group, and the peaks at 8.57(1H, s) can be assigned to the 2, 4-diamino-6-pteridinyl group of MTX as Fig. 2b suggests. The NMR of CS-MTX proposed the CS (disaccharide part δH signals were between 3.10 and 5.20, with 3.93 assigned as the anomeric carbon) was successfully attached to MTX (chemical shift of benzoyl group was 8.62 and 6.93, and the methyl group was at 3.42). The NMR also proved that MTX was conjugated to CS.


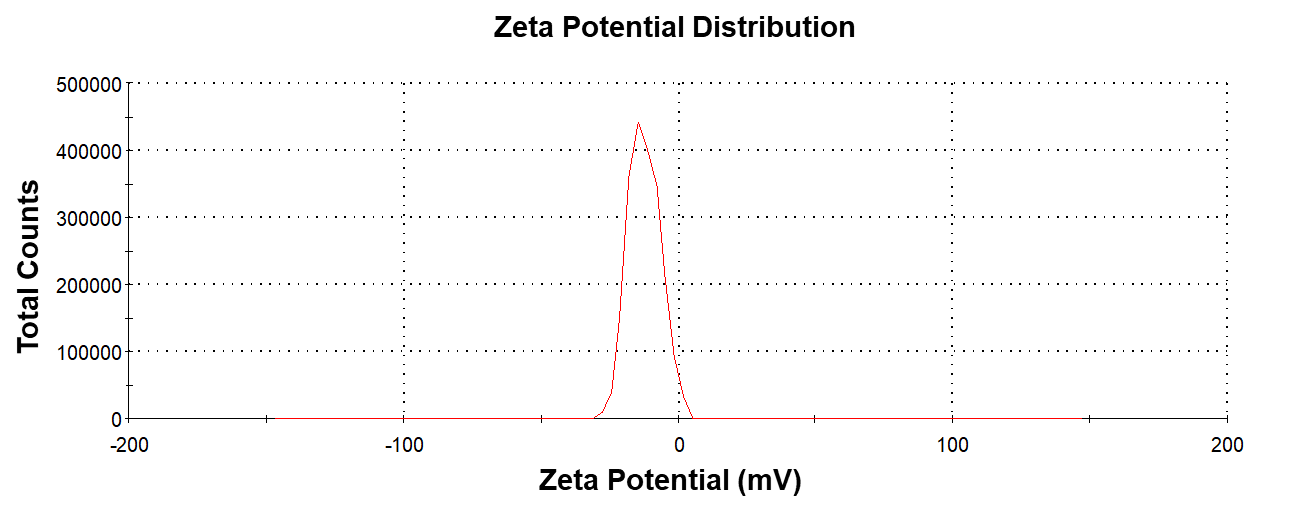


**Figure S3.** Zeta potential of C/M@Alb NCs dissolved in distilled water.


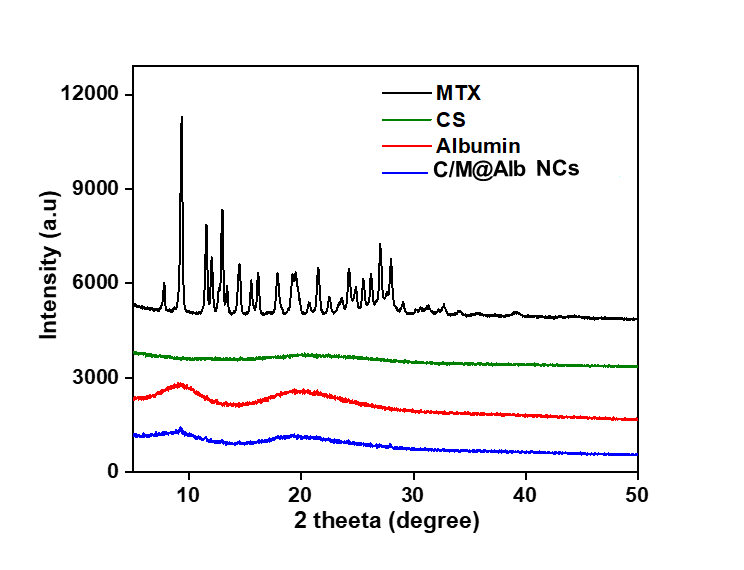


**Figure S4.** XRD analysis of C/M@Alb NCs in comparison to albumin, CS and MTX to analyze the nature MTX and CS after encapsulation ion albumin nanoparticles.


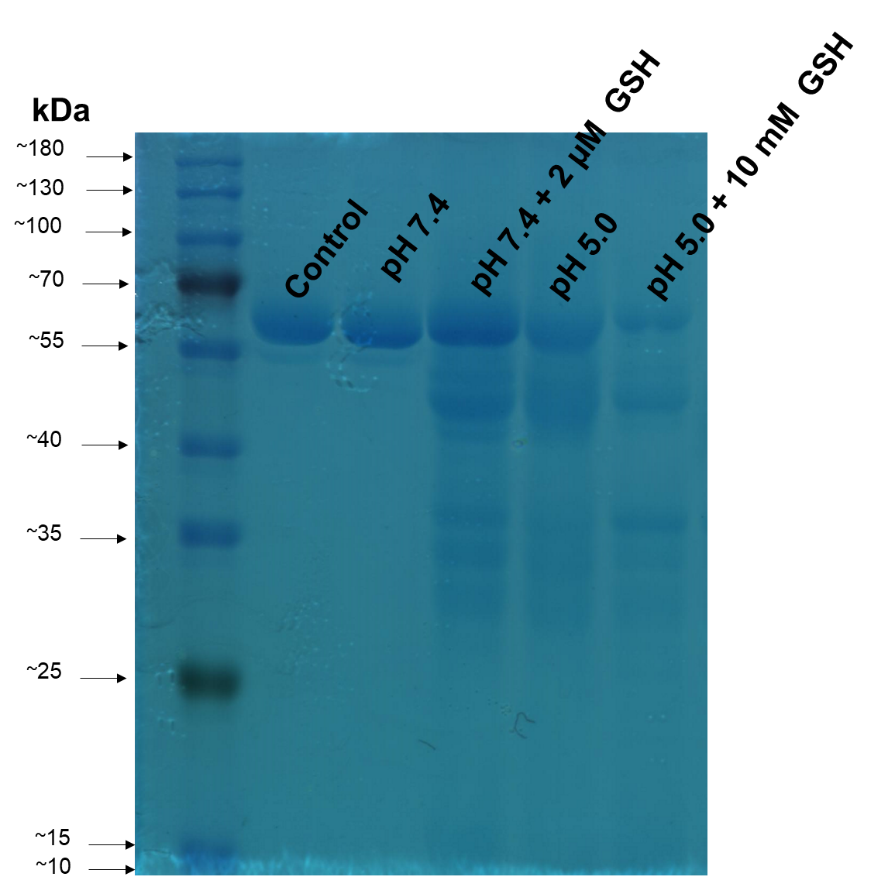


**Fig. S5.** SDS-PAGE of C/M@Alb NCs after incubation higher and low GSH concentration.


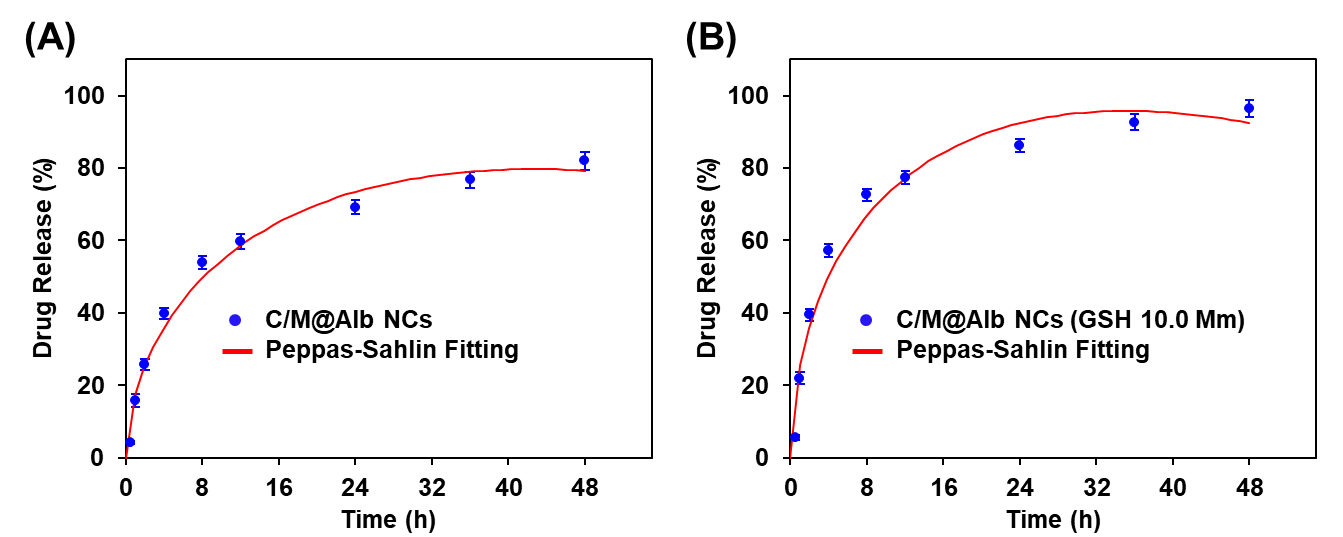


**Fig S6.** Graphical representation of the experimental data with Peppas-Sahlin fitting. (A) C/M@Alb NCs. (B) C/M@Alb NCs (GSH 10.0 mM).


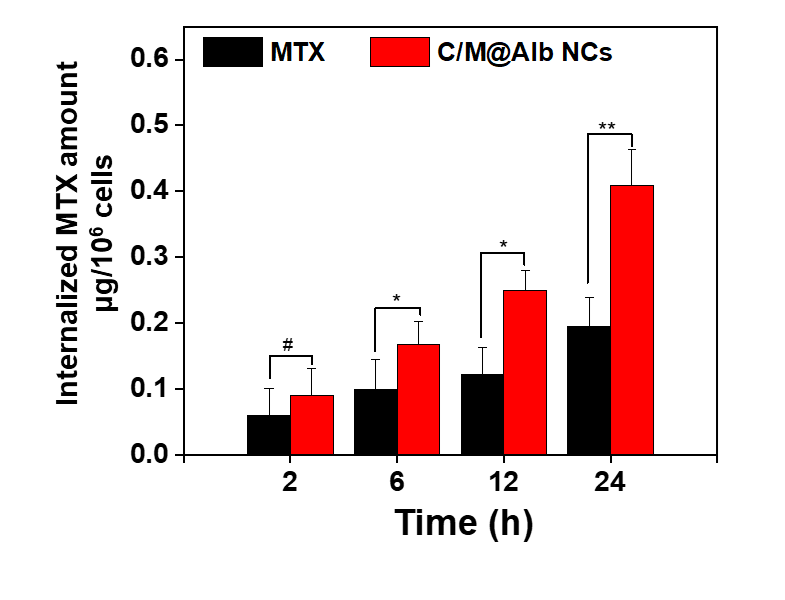


**Fig. S7.** Internalization of MTX in E0771 cancer cells treated with MTX and C/M@Alb NCs (2.0 µg mL^–1^) for 2, 6, 12 and 24 h.

**
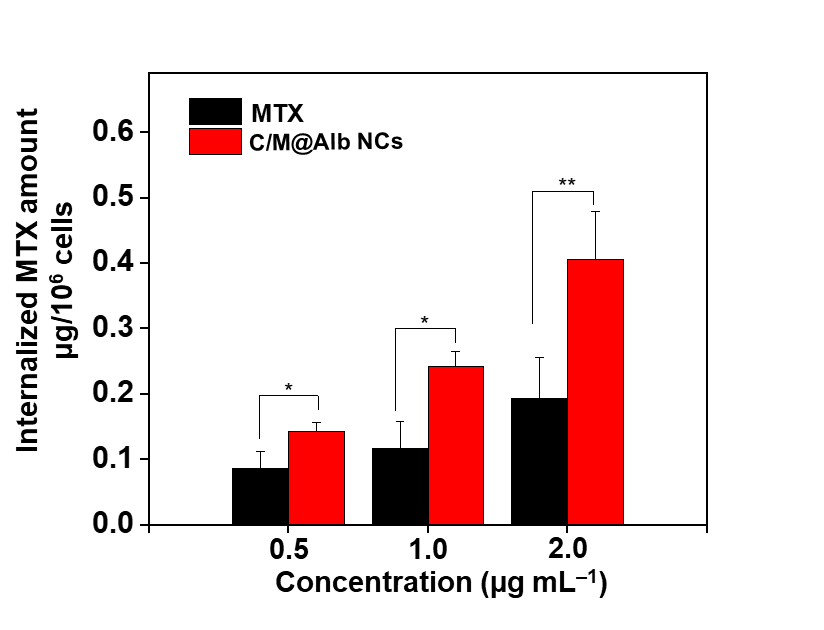
**

**Figure S8.** HT-29 cell-uptake of MTX and C/M@Alb NCs incubated at different concentrations of MTX (0.5-2 µg mL^–1^) for 24 h (*n* = 3). Data are expressed as mean ± SD. *p < 0.05, **p < 0.001 indicating significant statistical difference by student *t test*.


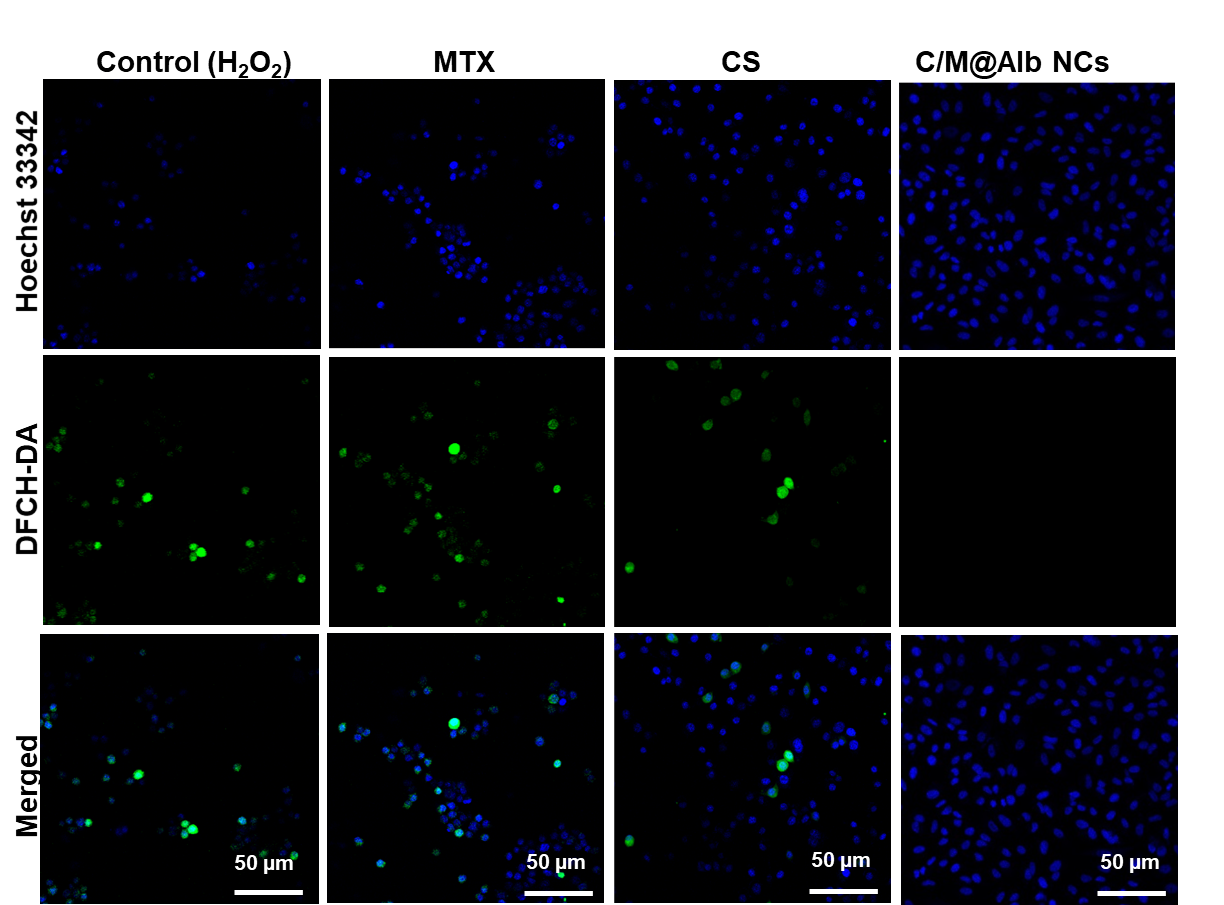


**Figure S9.** Intra-cellular ROS detection by DFCH assay using confocal microscopy after incubation with CS, MTX, C/M@Alb NCs (10.0 μg mL^–1^ CS, 5.0 μg mL^–1^ MTX) for 12 h.

**Figure S10.** Concentration-dependent intra-cellular ROS detection by DFCH assay using microplate reader. Data are expressed as mean ± SD. *p < 0.05, **p < 0.001 indicating significant and ^#^p > 0.05 is indicating in-significant statistical difference.

**
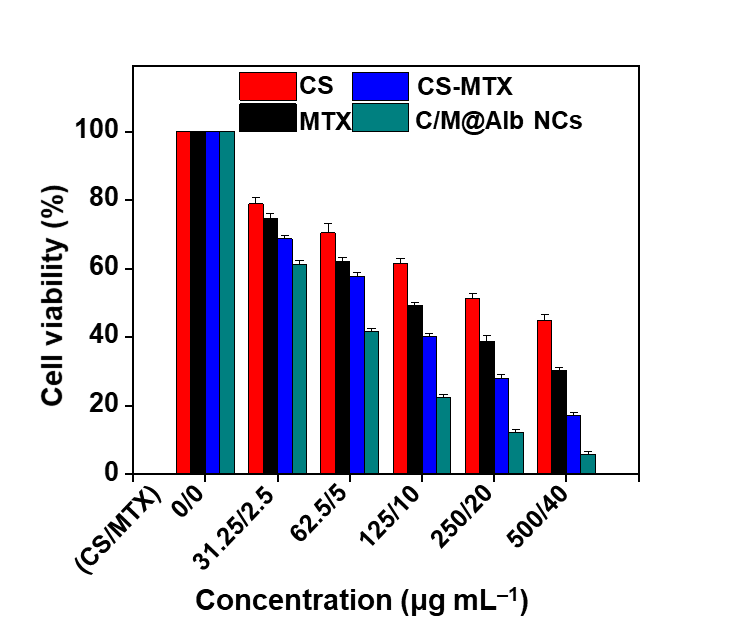
**

**Figure S11.** *In vitro* anti-tumor efficacy of C/M@Alb NCs against HT-29 tumor cells treated for 48 h. Data are expressed as mean ± SD.

**
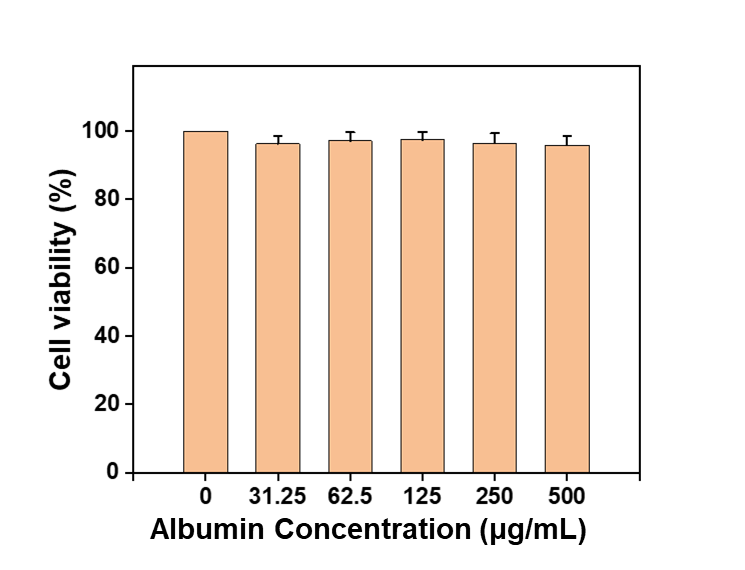
**

**Figure S12**. Effect of albumin on the viability (%) of HT-29 tumor cells for 24 h. Data are expressed as mean ± SD.


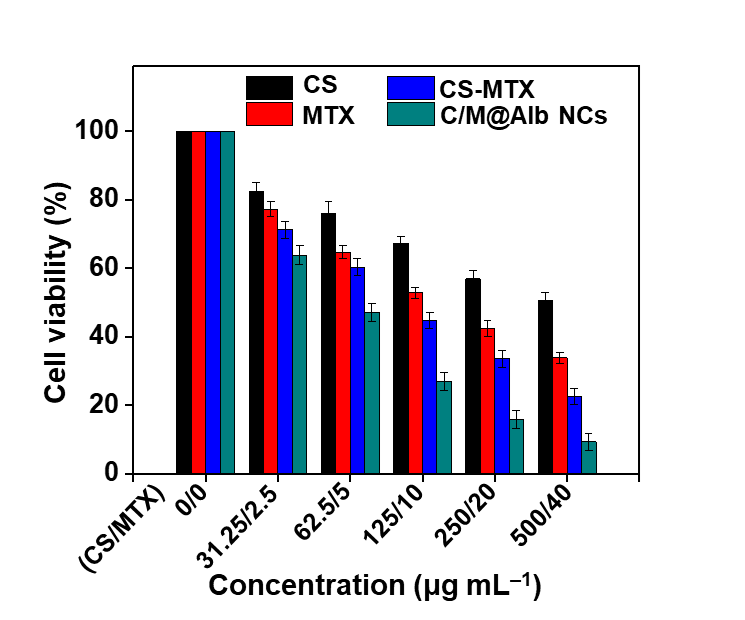


**Fig. S13.** Cell viability of E0771 cancer cells treated with C/M@Alb NCs and Free CS and MTX for 24 h.


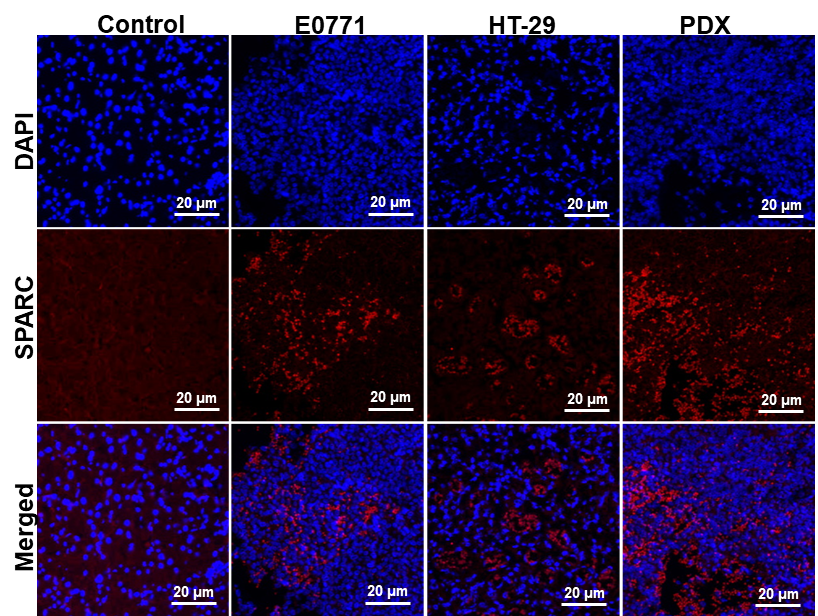


**Figure S14.** Immunofluorescence of SPARC (red) expression, nucleus (blue) and their colocalization in E0771 breast tumor, HT-29 colon tumor and PDX lung tumor. Mammary pad tissue was used as control.


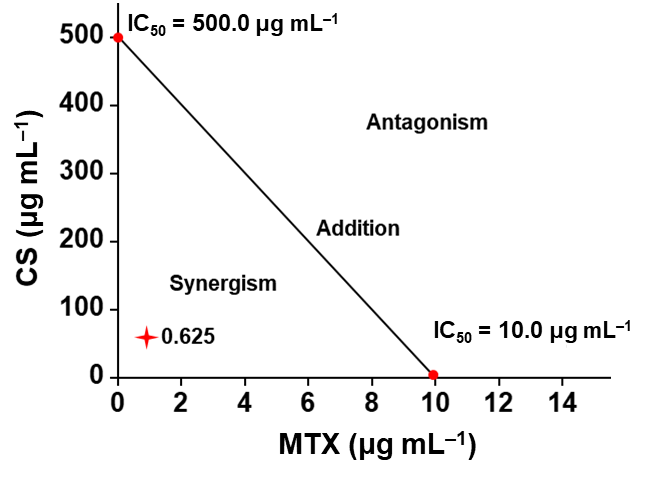


**Figure S15.** Combination index (CI) of CS and MTX (CI = 0.625).


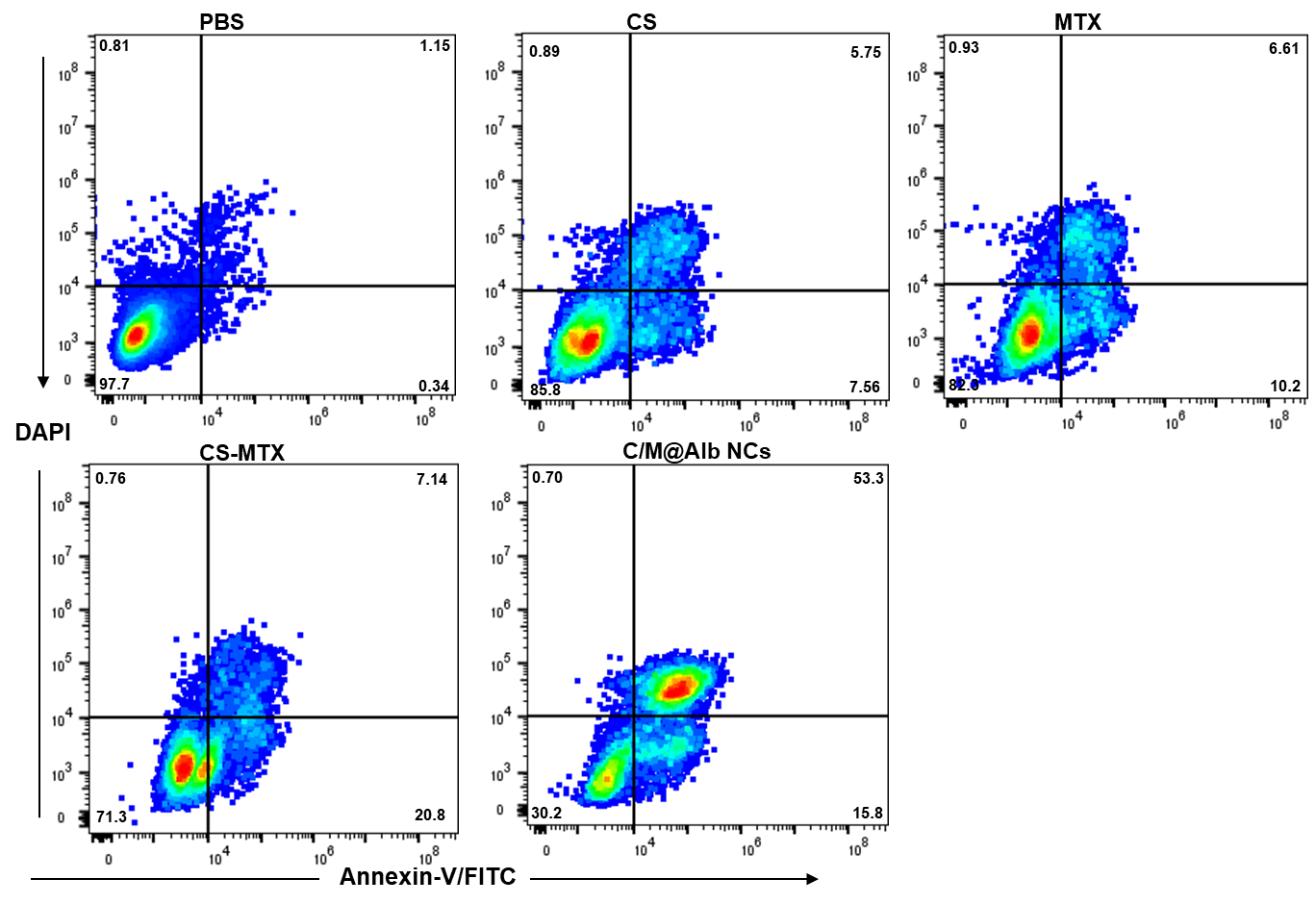


**Figure S16.** Effect of C/M@Alb NCs on cell apoptosis in colon cancer HT-29 cells. Colon cancer HT-29 cells were treated with PBS, CS, MTX, Combo of CS-MTX and C/M@Alb NCs for 24 h. The apoptosis rate (%) were calculated by cell apoptosis assay kit and flow cytometer.


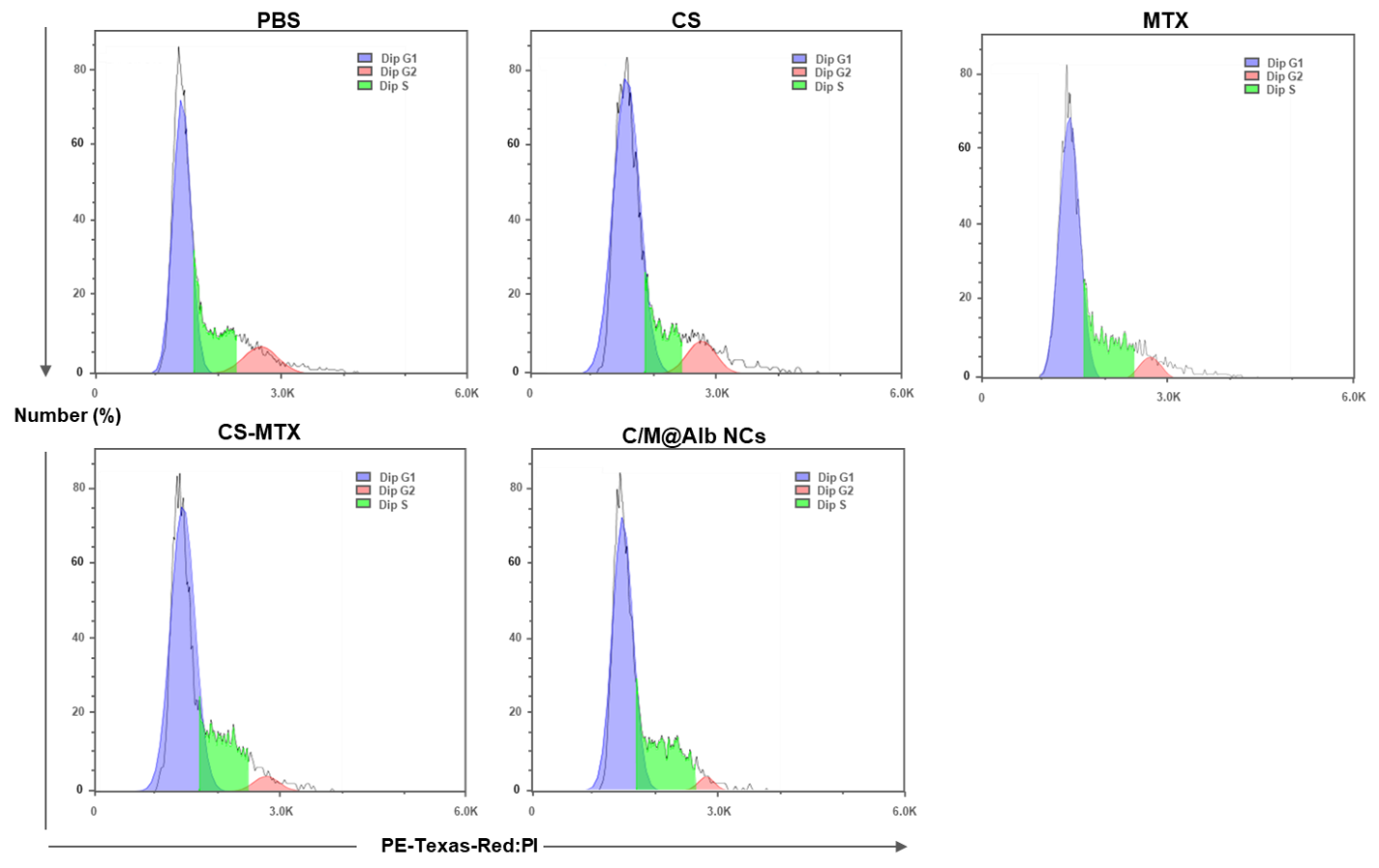


**Figure S17.** Effect of C/M@Alb NCs cell cycle arrest in colon cancer HT-29 cells. Colon cancer HT-29 cells were treated with PBS, CS, MTX, Combo of CS-MTX and C/M@Alb NCs for 24 h. The percentages of cells in each phase (G0/G1, S, G2/M) were calculated by cell cycle assay kit and flow cytometer.


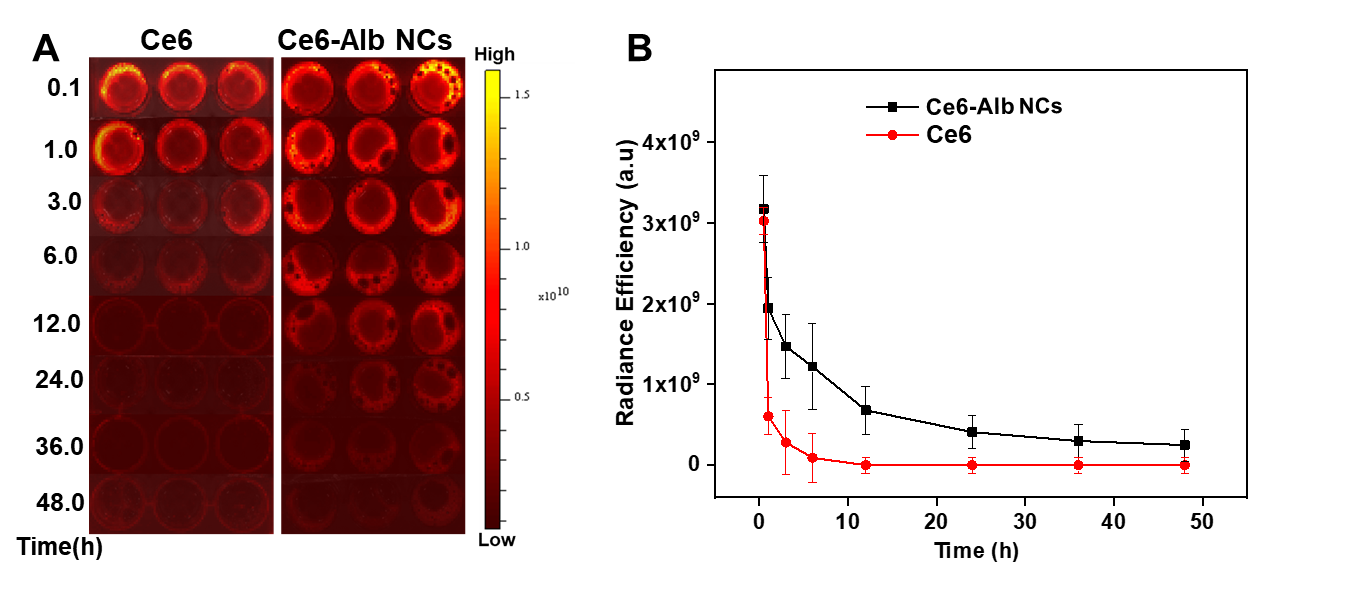


**Figure S18.** **(A)** NIRF images of blood samples from the mice treated with Ce6 AND Ce-conjugated albumin nanoparticles. **(B)** Quantification of fluorescence intensity. Data are expressed as mean ± SD.

**Figure S19.** Body weight of BALB/c C57BL/6 mice injected with C/M@Alb NCs and Free MTX doses (10.0 mg Kg^–1^, 20.0 mg Kg^–1^and 30.0 mg Kg^–1^).


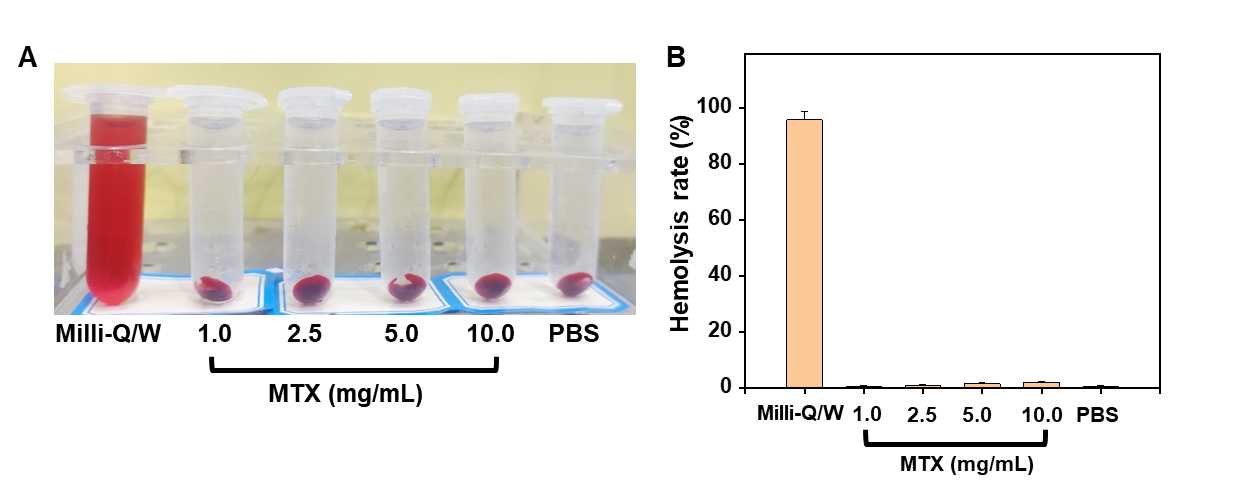
 **Figure S20.** Hemolysis of C/M@Alb NCs against mouse red blood cells. (A) Photos of centrifuged red blood cells after treatments of C/M@Alb NCs at different concentrations. (B) The hemolysis rates of different treatment groups. PBS and Milli-Q/W were used as the negative and positive controls, respectively.


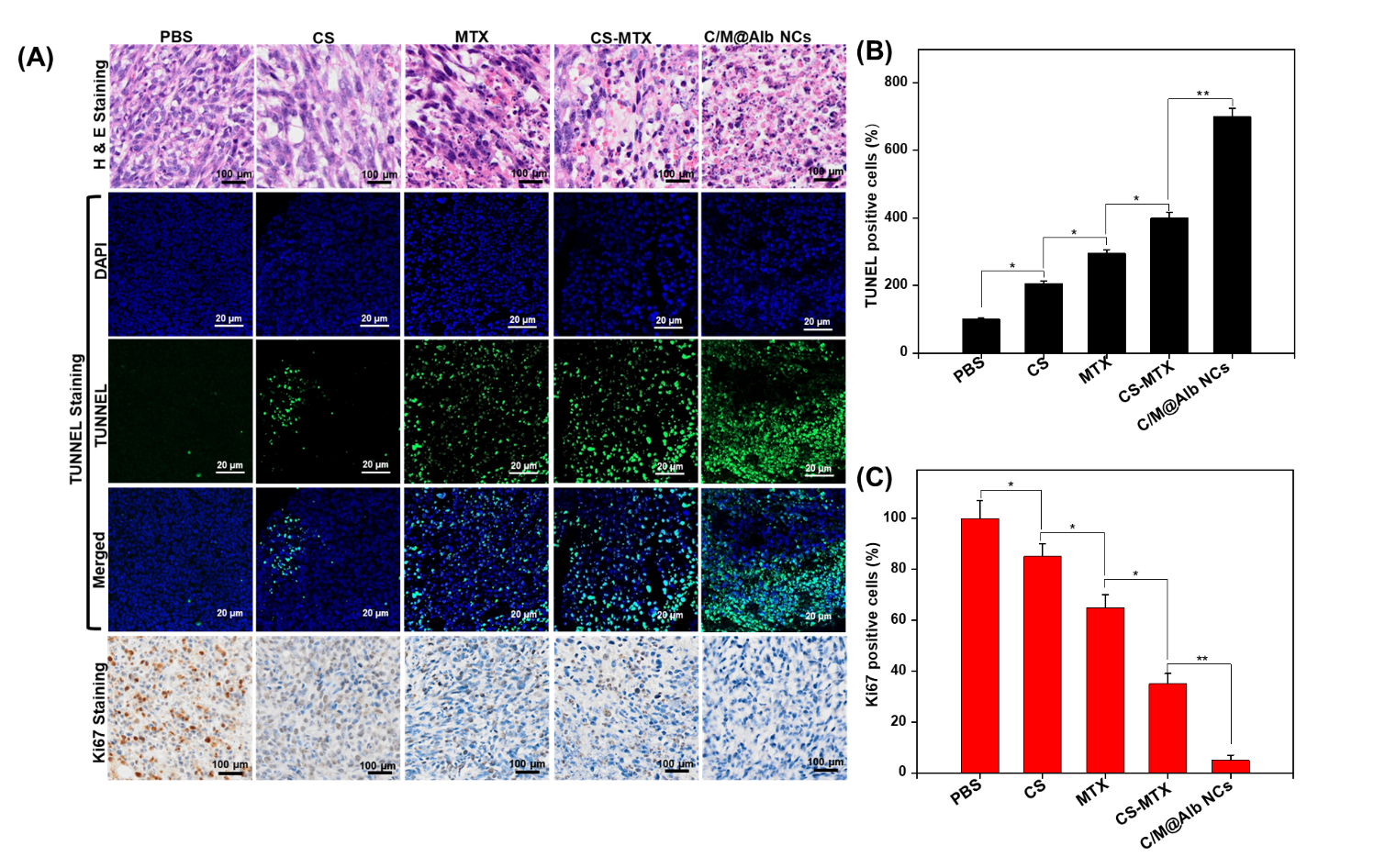


**Figure S21.** (A) *In vivo* tumor efficacy evaluation of C/M@Alb NPs by TUNEL assay, H&E and KI67 staining of tumor sections of the E0771 tumors in C57/BL6 mice treated with CS, MTX, CS-MTX and C/M@Alb NCs at the CS and MTX dose of ≈100.0 and 10.0 mg Kg^–1^, respectively. (B) TUNEL positive cells. (C) KI67 positive cells. Data are expressed as mean ± SD (n=5). * p < 0.05, ** p < 0.01 indicate a statistically significant difference (by Student *t*-test).


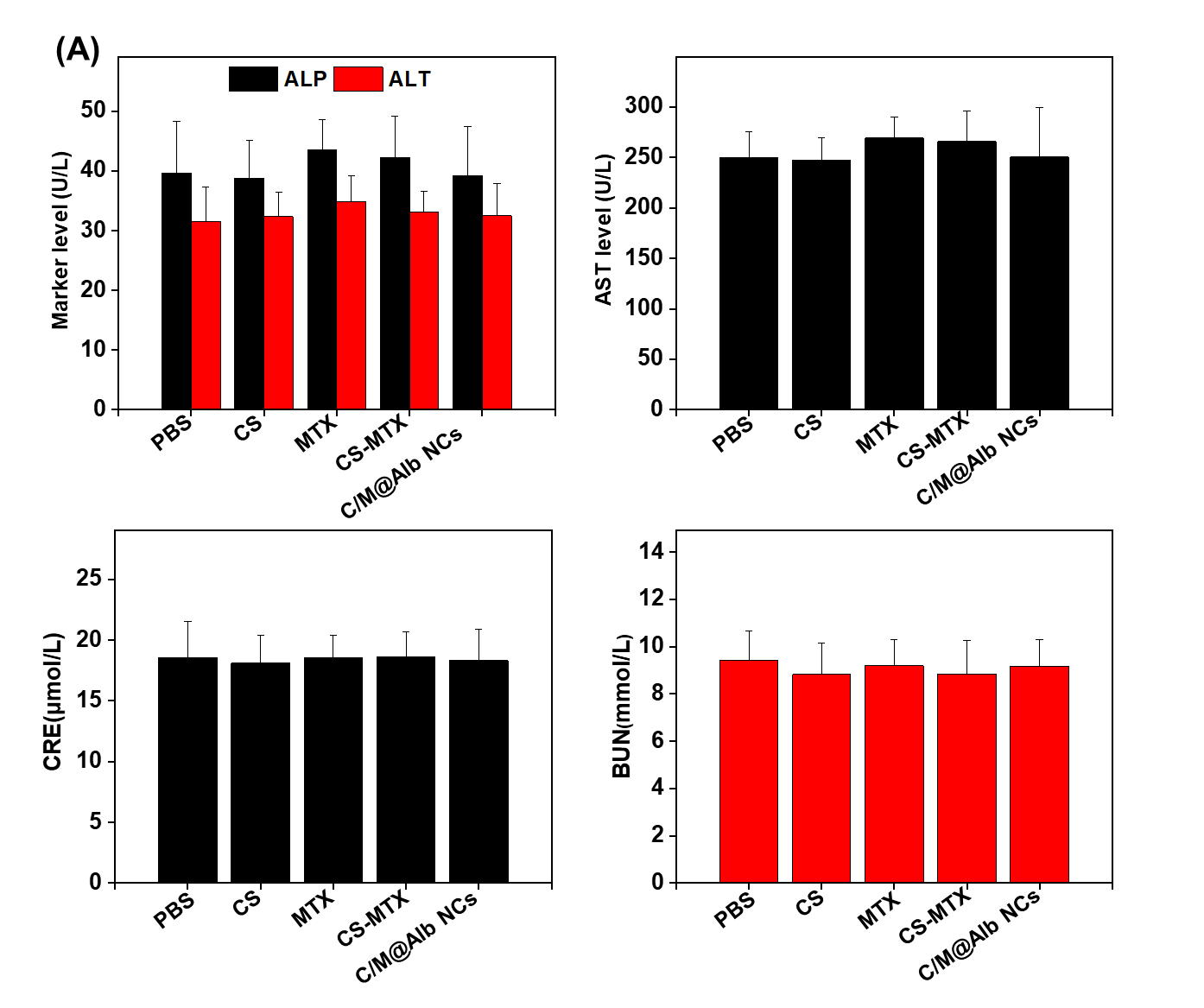


**Figure S22.** *In vivo* safety evaluation of free drugs (CS, MTX, CS-MTX) and C/M@Alb NCs (100.0 mg Kg^–1^ CS and 10.0 mg Kg^–1^ MTX) in HT-29 tumor-bearing nude BALB/c mice (N=25, n=5/Group). (A) Level of ALP and ALT. (B) Level of AST. (C) Level of CRE. (D) BUN level. Data are expressed as mean ±SD (n=5).


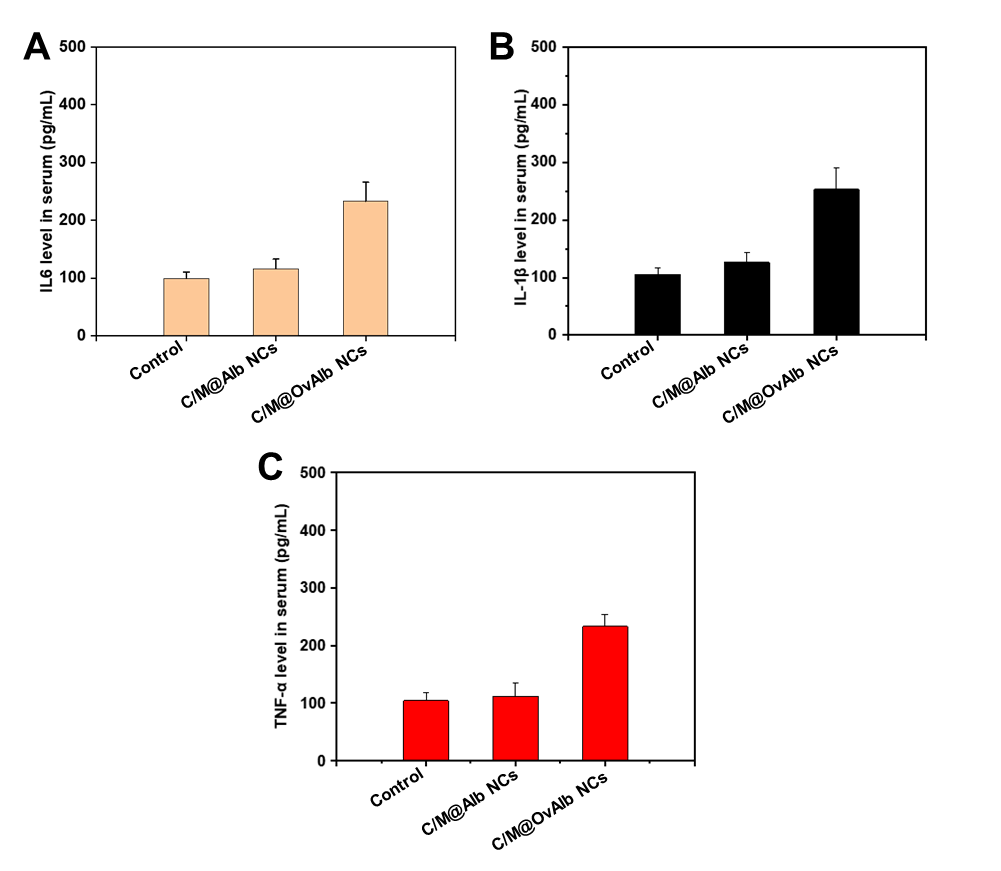


**Fig. S23.** Enzyme-linked immunosorbent assay (ELISA) measurement of cytokines level (A) IL-6 (B) IL-1β (C) TNF-α in mouse blood serum.

**Table S1. Accelerated stability of C/M@Alb NCs**

| **Temperature (°C)** | **Observation** | | | |
| --- | --- | --- | --- | --- |
|  | **Morphology** | | **Particle size** | **Drug content** |
| -20 | - | - | | - |
| 4 | - | - | | - |
| 25 | + | + | | - |
| 37 | + | + | | + |

**Table S2.** Fitting of the drug release profile of C/M@Alb NCs to the Peppas–Sahlin model.

| **Samples** | **k1** | **k2** | **n** | **R^2^** |
| --- | --- | --- | --- | --- |
| C/M@Alb NCs | 18.82 | -1.11 | 0.56 | 0.990 |
| C/M@Alb NCs (GSH) | 28.11 | -2.06 | 0.54 | 0.981 |

**Table S3. Pharmacokinetic parameters of C/M@Alb NCs in comparison to CS and MTX**

| **Parameters** | **t_1/2β_ (h)** | **AUC (µg/mL) ×h** |
| --- | --- | --- |
| MTX | 1.5 | 7.91 |
| C/M@Alb NCs | 5.78 | 90.5 |
